# LogoXpertNet: a novel lightweight logo classification using deep learning

**DOI:** 10.1038/s41598-026-45682-z

**Published:** 2026-03-26

**Authors:** Muhammad Tahir Mumtaz, Mohd Khalid Awang, Muhammad Usman Saeed, Shafiq Hussain, Dhouha Ben Noureddine, Yassine Daadaa

**Affiliations:** 1 Faculty of Informatics and Computing, Universiti Sultan Zainal Abidin (UniSZA), Terengganu, Malaysia; 2 School of Computer Science and Technology, Harbin Institute of Technology, Shenzhen, 518055 China; 3 Department of Computer Science, University of Sahiwal, Sahiwal, Punjab Pakistan; 4 College of Computer and Information Sciences, Imam Mohammad Ibn Saud Islamic University (IMSIU), 13318 Riyadh, Saudi Arabia

**Keywords:** Logo classification, Lightweight, Deep learning, Feature fusion, Computational biology and bioinformatics, Engineering, Mathematics and computing

## Abstract

Logo classification is crucial in various applications, including brand monitoring, copyright protection, and digital forensics. Traditional computer vision techniques face significant limitations, particularly in handling scale variations, occlusions, and background clutter. While deep learning models, particularly convolutional neural networks (CNNs), offer superior solutions, they often come with high computational costs, posing challenges for real-time deployment. This paper introduces LogoXpertNet, a lightweight deep learning architecture specifically designed for efficient logo classification. The key innovations of LogoXpertNet include: (1) a modified MobileNetV3 backbone enhanced with bottleneck and squeeze-and-excitation (SE) blocks for efficient feature extraction; (2) a novel cross-layer feature fusion (CLFF) module that improves feature integration across network depths; (3) a newly proposed hierarchical squeeze-excitation spatial attention block (HSE-SAB) that dynamically attends to both spatial and channel-wise features; and (4) a feature-aware convolution block attention module (FA-CBAM) that uniquely fuses spatial and frequency-domain information for refined logo attention. Extensive experiments on the evaluated benchmark datasets, including FlickrLogos-32, BelgaLogos, and WebLogo-2M, show that LogoXpertNet achieves strong classification performance while maintaining low computational overhead. Because the reported accuracies approach saturation on these benchmarks, the results should be interpreted in the context of dataset characteristics, split construction, and metric definition rather than as evidence of universal performance across all real-world logo-recognition scenarios. LogoXpertNet provides an efficient and practical framework for benchmark logo classification and offers a promising basis for future validation under more challenging real-world conditions.

## Introduction

Logo classification play a crucial role in a variety of real-world applications, including brand monitoring, copyright protection, market analytics, and augmented reality. With the rapid growth of digital media and e-commerce platforms, billions of images and videos are uploaded daily across the internet, many of which contain branded content^1^. Identifying these logos manually is impractical, which has led to increased interest in automated systems capable of recognizing logos accurately in diverse environments. Traditional computer vision techniques, although effective in constrained settings, struggle with variations in scale, occlusion, lighting, and background clutter^2^.

Deep learning has emerged as a powerful solution to overcome these challenges due to its ability to automatically learn hierarchical representations from raw data. Convolutional Neural Networks (CNNs) and modern classification frameworks such as Faster R-CNN, YOLO, and SSD have revolutionized object detection by significantly improving accuracy and inference speed. When applied to logo recognition, these models can not only localize logos within images but also classify them into their corresponding brand categories. Additionally, transfer learning enables the use of pre-trained models on large-scale datasets, reducing the need for vast amounts of labeled logo data and accelerating development time^3^.

Despite these advancements, logo classification presents unique challenges that differentiate it from general object classification tasks. Logos often appear in distorted forms, printed on fabric, embedded within videos, or partially occluded. Furthermore, the high intra-class variation and low inter-class variation between certain brand logos make classification difficult^4^. Developing robust models requires careful consideration of data augmentation strategies, anchor box settings, and domain adaptation techniques to ensure generalization across diverse environments and platforms^5^.

However, many significant research gaps persist. First, while existing models deliver high accuracy, they often come with substantial computational costs, making real-time deployment on resource-constrained devices challenging^6,7^. Second, many approaches lack efficient mechanisms to simultaneously handle multi-scale features, channel spatial attention, and frequency-domain information in a unified, lightweight architecture^8,9^. Third, although attention modules like SE and CBAM exist, they are not specifically optimized for the unique characteristics of logos, such as high intra-class variation, stylized text, and complex background interactions^10^. To bridge these gaps, a lightweight, efficient, and highly accurate model is needed, one that maintains low computational overhead without sacrificing performance in diverse real-world conditions. This requires a novel architecture that integrates multi-scale feature fusion, hierarchical attention, and frequency-aware mechanisms specifically designed for logo semantics.

To address these needs, the authors propose LogoXpertNet, a novel deep learning architecture that incorporates a modified MobileNetV3 backbone for efficient feature extraction, utilizing bottleneck and Squeeze-and-Excitation (SE) blocks to optimize computational efficiency. A Cross-Layer Feature Fusion (CLFF) module is introduced to enhance feature integration between early and deep layers, improving the model’s ability to handle logos of varying complexities. Additionally, the Hierarchical Squeeze-Excitation Spatial Attention Block (HSE-SAB) is designed to dynamically focus on important spatial and channel-wise features, enhancing classification in diverse environments. The final component, the Feature-Aware Convolution Block Attention Module (FA-CBAM), fuses spatial and frequency-domain information to refine the model’s attention toward logo-specific structures. This multi-stage architecture not only improves logo recognition performance but also adapts well to real-world challenges like scale variation, background clutter, and occlusion. The main contributions of this approach are:

- To introduce LogoXpertNet, a lightweight deep learning model for efficient logo classification.
- To develop the CLFF module to enhance feature integration from multiple network layers.
- To propose the HSE-SAB to improve the model’s focus on both spatial and channel dimensions for logo classification.
- To introduce the FA-CBAM, combining spatial and frequency-domain features to improve logo classification in complex conditions.
- To conduct extensive experiments on benchmark datasets and to analyze the proposed model’s performance under the specific benchmark settings used in this study, while acknowledging that additional validation is still needed for broader real-world deployment scenarios.

Unlike generic object categories, logos often exhibit high intra-class variation combined with low inter-class discrimination, where visually distinct instances of the same brand coexist alongside highly similar logos from different brands. Existing attention mechanisms such as SE and CBAM rely primarily on global pooling and single-layer recalibration, which can suppress fine-grained structural details and repetitive high-frequency patterns that are characteristic of logos, including stylized text, sharp contours, and geometric motifs. To address these limitations, LogoXpertNet is explicitly designed to preserve and emphasize logo-specific cues through cross-layer feature fusion, hierarchical multi-scale attention, and frequency-aware enhancement, enabling robust discrimination under scale variation, occlusion, and background clutter.

## Related work

Logo classification has become an essential task in applications such as brand monitoring, copyright protection, advertisement analysis, and augmented reality. Traditional computer vision techniques often struggle with challenges such as scale variation, occlusion, background clutter, and diverse logo designs. With the rapid advancement of deep learning, CNNs and transformer-based architectures have demonstrated remarkable performance in object classification tasks, enabling more accurate and robust logo localization and classification. As a result, deep learning–based approaches have emerged as the state-of-the-art solution for reliable and scalable logo classification. Brand logos are crucial visual identities for businesses, driving research in logo recognition and analysis within computer vision. Traditional methods are inefficient, leading to the development of automatic recognition systems. This study introduces a logo identification and analysis system using CNN^11^, achieving a recognition accuracy of 95.8% on 10,000 logos and 92.3% accuracy in analyzing design elements like color and shape. While the system outperforms traditional methods, it has limitations, including reduced accuracy with logos featuring complex backgrounds or unusual designs, and reliance on large, annotated datasets for training.

Logo detection involves locating and classifying logos in images and videos, with many existing methods relying on general object classification approaches that fail to fully capture the unique characteristics of logos, resulting in sub-optimal performance. Recent advancements have introduced the Context-based Modeling Enhancement Network (CME-Net)^12^, which incorporates contextual information from both the logo and its background to enhance the saliency of distinctive regions. A scale feature balance strategy is also employed to preserve scale information and suppress noise generated during the enhancement process. Experimental results on four benchmark datasets demonstrate that CME-Net significantly improves logo classification accuracy, particularly in complex environments. However, a notable limitation is that performance may still be compromised by logos with intricate backgrounds or severe occlusions.

Li et al.^13^ proposes a vehicle logo classification method using the Swin Transformer, which leverages hierarchical vision transformers with shifted-window attention to balance local and global feature extraction. Tested on three public datasets (HFUT-VL1, XMU, CTGU-VLD), the approach achieved near-perfect accuracy and outperformed CNNs and ViT in both accuracy and speed. However, limitations include reliance on large-scale labeled datasets, potential challenges with highly imbalanced classes, and the need for further validation in real-world, diverse traffic environments where lighting, occlusion, and motion blur may reduce performance.

For data-efficient learning, Sareer et al. proposed a deep learning-based active learning (DLBAL) method that intelligently selects both uncertain and high-confidence samples using EfficientNet-B0, significantly reducing manual labeling effort for image classification on datasets like CACD and Caltech-256. For video surveillance^14^, high-performance anomaly classification models were developed, including an attention-based framework (ADSV)^15^, a 3D CNN-LSTM^16^ with novel feature fusion, and the compact EADN model^17^. These systems combine CNNs and LSTMs to effectively capture spatiotemporal features, achieving state-of-the-art accuracy with minimal model size and computational cost on benchmark datasets such as UCF-Crime, CUHK-Avenue, and UCSD Pedestrian.

### Logo classification using FlickrLogos-32 dataset

Detecting logos in real-world environments remains challenging due to variations in scale, lighting, occlusion, and background complexity. The FlickrLogos-32 dataset, comprising images collected from natural scenes with significant intra-class variability and cluttered backgrounds, serves as a widely adopted benchmark for evaluating logo classification algorithms. With the advent of deep learning, particularly CNN-based object detectors such as Faster R-CNN, YOLO, and SSD, the performance of logo classification systems on datasets like FlickrLogos-32 has improved significantly. These models enable end-to-end learning of robust visual features, allowing for accurate localization and classification of logos even in unconstrained conditions. Therefore, leveraging deep learning on the FlickrLogos-32 dataset provides an effective pathway toward developing scalable and reliable logo recognition systems for real-world applications.

Sujini et al.^18^ presented an automated logo classification system utilizing deep learning models. The system addresses challenges in classifying logos within complex, real-world images by leveraging transfer learning, data augmentation, and synthetic data generation. The approach achieves high accuracy in logo classification, with applications in brand visibility analysis and media monitoring. The study compares different architectures and training strategies to optimize performance, demonstrating the effectiveness of deep learning techniques in logo classification tasks.

Hubenthal et al.^19^ presented a novel approach to open-set logo recognition by leveraging multimodal image-text pre-training, primarily using Vision Transformer (ViT) models trained with contrastive loss functions like CLIP. The method enhances the model’s sensitivity to textual content within logos, enabling improved classification performance without additional OCR modules. Key innovations include the use of large-scale image-text datasets for pre-training and a new metric learning loss, ProxyNCAHN++, which incorporates class-specific hard negatives to improve class separation and robustness. The system pipeline involves checking logo regions, extracting text-aware embeddings, and matching them against a dynamic database. Experimental results demonstrate state-of-the-art performance, especially on text-heavy logos, with significant improvements over previous methods. The approach highlights the effectiveness of multimodal pre-training and specialized loss functions in advancing logo recognition. While also classifying challenges related to small, blurry, or stylized logos.

Fujitake et al.^5^ introduced RL-LOGO, a deep reinforcement learning-based approach for logo classification that does not require explicit positional annotations. Using a deep Q-network (DQN) and a confidence-guided reward function, the method iteratively classifies logos in images. Experimental results on multiple benchmark datasets demonstrate that RL-LOGO achieves state-of-the-art accuracy, effectively handling challenges like scale variation and background noise, and showing strong potential for real-world applications. Shulgini et al.^20^ presents a scalable, zero-shot logo recognition system that combines a universal logo classifier based on Scaled-YOLOv4 with an enhanced CLIP model for zero-shot classification. The approach efficiently classifies them without additional training, achieving high accuracy on datasets like FlickrLogos-32. The system outperforms traditional methods in speed and accuracy, demonstrating its potential for real-world, large-scale logo classification and recognition applications. Key contributions include establishing a new baseline for logo classification and showcasing the effectiveness of single-stage detectors combined with zero-shot classifiers.

Table 1 shows the summary of the SOTA methods for the FlickrLogos-32 dataset.

**Table 1:** Summary of SOTA methods on logo classification using the FlickrLogos-32 dataset.

Reference	Approach	Key contributions	Disadvantages
Sujini et al.^18^	Deep learning	Transfer learning, data augmentation	High computational cost, requires large labeled datasets for training
Hubenthal et al.^19^	ViT with multimodal pre-training	Text-aware logo recognition	High resource consumption, slower training due to large datasets
Fujitake et al.^5^	Deep reinforcement learning	No need for positional annotations, handles scale variation	High computational demand, slower convergence in complex scenarios
Shulgin et al.^20^	Scaled-YOLOv4 and CLIP for zero-shot recognition	No training needed, fast inference, new detection baseline	Requires high-quality datasets, computationally expensive during inference

### Logo classification using BelgaLogos dataset

Logo classification is essential for applications like brand monitoring and visual content analysis. The BelgaLogos dataset, which contains real-world news images with annotated logos, offers a challenging benchmark due to variations in size, orientation, and background clutter. Deep learning models such as Faster R-CNN and YOLO have shown strong performance on this dataset by learning robust visual features that enable accurate localization and identification of logos. As a result, deep learning has become a reliable solution for handling complex logo recognition tasks.

Abyaa et al.^21^ presented LogoTrust, a novel dataset of brand logos linked to domain names, constructed using BIMI DNS records. By performing large-scale DNS measurements, the authors collected, de-duplicated, and validated over 1800 logos across more than 2800 domains, demonstrating BIMI’s potential for high-integrity logo datasets. The methodology involves verifying certificates and aggregating logos, with applications in phishing detection and brand security. The study highlights the advantages of using BIMI for dynamic, reliable logo collection while acknowledging limitations such as BIMI adoption rates and logo variability. The dataset is publicly available to support further research in brand verification and cybersecurity.

Boia et al.^22^ introduced a robust logo classification method that combines homography matching of SIFT keypoints, graph-based modeling, and multiple instance learning to effectively detect and classify logos in natural images. The approach addresses challenges such as perspective distortion, shape and color variations, occlusions, and background clutter. Key innovations include the use of weighted homography graphs, secondary models for color-inverted logos, and strategic image upscaling for small logos. Evaluated on datasets like FlickrLogos-32 and BelgaLogos, the method achieves over 90% recognition accuracy, outperforming existing techniques, though it still faces challenges with very small, symmetrical, or heavily occluded logos.

Chu et al.^23^ presented a logo classification system that leverages local features (such as SIFT), visual word histograms, and spatial visual patterns to identify logos in large datasets like BelgaLogos and FlickrLogos. The approach combines feature matching, spatial relationship analysis, and clustering techniques to accurately detect logos, achieving high overlap ratios and outperforming some existing methods. The system demonstrates robustness to scale, rotation, and small distortions, with future work aimed at improving performance for logos with fewer features and developing new features. Table 2 shows the summary of the SOTA methods for the BelgaLogos dataset.

**Table 2:** Summary of SOTA methods on logo classification using the BelgaLogos dataset.

Reference	Approach	Key contributions	Disadvantages
Abyaa et al.^21^	BIMI DNS records for logo dataset	Large-scale DNS measurement for logo collection, applied in phishing detection	High data collection cost, dependent on BIMI adoption, limited logo variability
Boia et al.^22^	Homography matching, SIFT keypoints, graph-based modeling	Achieves over 90% accuracy, handles occlusions, color variations, and small logos	Computationally expensive due to keypoint matching and graph processing, limited by occlusion
Chu et al.^23^	Local features (SIFT), visual word histograms, clustering	High robustness to scale, rotation, and small distortions, outperforms some methods	Computationally intensive with feature matching, clustering, and requires large datasets

### Logo classification using WebLogo 2M

The WebLogo-2M dataset has emerged as a valuable large-scale benchmark for logo classification, offering over two million real-world images collected from the web with noisy annotations. Unlike earlier curated datasets such as FlickrLogos-32 or LogoDet-3K, WebLogo-2M introduces considerable variability in logo appearance due to diverse backgrounds, occlusions, scale differences, and image compression artifacts, making it ideal for training robust deep learning models. Recent studies have leveraged WebLogo-2M to train region-based and one-stage detectors, such as Faster R-CNN and YOLO variants, demonstrating significant improvements in generalization to unseen data. Furthermore, the dataset’s inherent label noise has encouraged the development of weakly supervised and noise-tolerant learning strategies, positioning WebLogo-2M not only as a dataset for benchmarking performance but also as a testbed for real-world logo recognition challenges.

Wang et al.^24^ presented a Logo-2K+, a large-scale, publicly available dataset containing 2341 logo categories and 167,140 images, designed to facilitate scalable logo classification. To improve logo classification accuracy, a deep learning framework was proposed with four key components: a navigator for coarse region proposals, a teacher for confidence evaluation, a region-oriented data augmentation module, and a scrutinizer for feature fusion and final prediction. The model employs a coarser-to-finer localization strategy to identify discriminative logo regions effectively. Extensive experiments on datasets such as Logo-2K+, BelgaLoges, FlickrLogo-32, and WebLogo-2M show that DRNA-Net outperforms existing methods, achieving higher Top-1 and Top-5 accuracies. For example, on WebLogo-2M, DRNA-Net achieved a Top-1 accuracy of approximately 65%, surpassing baseline models like ResNet-50. Visualization results confirm its ability to accurately localize relevant logo regions, demonstrating the effectiveness of region-based localization and data augmentation strategies in large-scale logo recognition.

Su et al.^25^ introduced SL2 (Scalable Logo Self-co-Learning). It tackles scalable logo classification by learning from noisy web data without manual bounding-box labeling. Key ideas include incremental self-co-learning, where two detectors (Faster R-CNN and YOLOv2) mutually improve through self-mining of high-confidence detections; cross-model co-learning to leverage complementary strengths; and context-enhanced data to address class imbalance. The paper presents WebLogo-2M, a large web-derived logo dataset (about 2.19 million images across 194 classes). SL2 demonstrates substantial gains over fully supervised, weakly supervised, and weakly supervised baselines, showing that self-/co-learning with synthetic, context-rich data enables scalable logo classification and easy dataset expansion for new classes.

Sahel et al.^26^ review the main methods along with domain-specific techniques. It covers datasets like FlickrLogos-32, WebLogo-2M, and PL2K to evaluate performance. A central finding is that applying pretrained detectors with transfer learning substantially reduces labelling costs while delivering strong results on benchmark datasets; one- and few-shot learning setups can further boost efficiency and accuracy. The study notes trade-offs: two-stage classifiers tend to achieve higher accuracy at the expense of speed and compute, whereas single-stage detectors offer faster inference with competitive performance. The CNN-based LD is effective, with performance influenced by scale, viewpoint, and dataset characteristics, and future directions include exploring newer architectures and balancing accuracy with training and development costs. Table 3 shows the summary of the SOTA methods for the WebLogo-2M dataset.

**Table 3:** Summary of SOTA methods on logo classification using the WebLogo-2M dataset.

Reference	Approach	Key contributions	Disadvantages
Wang et al.^24^	DRNA-Net with coarse-to-fine localization	Outperforms existing models, achieves high accuracy on WebLogo-2M, effective region-based localization	High computational cost for training, requires large-scale datasets, slow inference
Su et al.^25^	SL2 self-/co-learning with noisy web data	Scalable logo classification without manual bounding-box labeling, addresses class imbalance	High data complexity, dependent on noisy web data, resource-intensive for co-learning
Sahel et al.^26^	R-CNN variants, RetinaNet, YOLO, DenseNet	Transfer learning reduces labeling costs, fast inference with single-stage detectors, high accuracy	Trade-offs in accuracy vs speed, higher resource consumption with two-stage detectors

## Methodology

### Proposed LogoXpertNet for logo classification

**Fig. 1:**
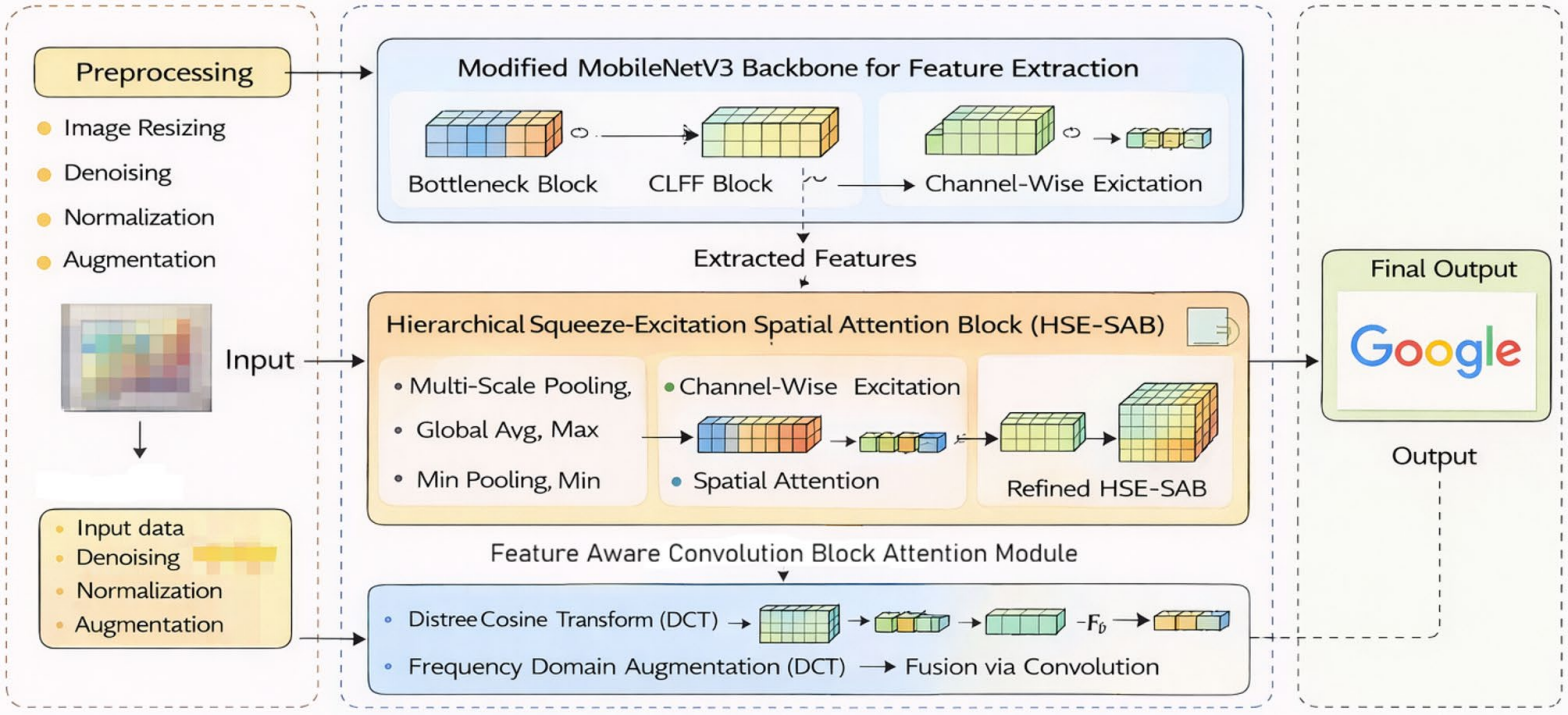
The architecture of the proposed model for logo classification.

The proposed LogoXpertNet utilizes a comprehensive deep learning architecture for image classification and is shown in Fig. 1. First, a modified MobileNetV3 model is used for feature extraction from the input dataset. This model integrates bottleneck blocks and modified Squeeze-and-Excitation (SE) blocks to optimize computational efficiency and channel-wise feature adaptation. Further, the HSE-SAB block is employed to extract multi-scale features by applying average, max, and min pooling, followed by excitation and fully connected layers. It is refined using spatial attention and sigmoid activation. Additionally, the Feature-Aware Convolutional Block Attention Module (CBAM) enhances feature representation by employing channel and spatial attention mechanisms, alongside a Discrete Cosine Transform (DCT) for further refinement. The final output is obtained after processing through a fully connected layer, enabling the model to classify the input images effectively. This architecture combines efficient feature extraction with advanced attention mechanisms, ensuring superior performance in image recognition tasks.

The proposed methodology begins with a preprocessing stage that includes image resizing, denoising, normalization, and augmentation to prepare input data. Feature extraction is then performed using a modified MobileNetV3 backbone, which incorporates Bottleneck Blocks for efficiency and a novel CLFF Block to integrate multi-level features. Following this, an HSE-SAB processes the extracted features through multi-scale pooling (average, max, and min), hierarchical channel-wise excitation using fully connected layers, and spatial attention via 2D convolution to dynamically focus on logo-relevant regions. Finally, an FA-CBAM refines the features by applying channel attention, spatial attention, and frequency-domain enhancement through DCT, which are fused and processed through convolutional and fully connected layers to produce the final refined feature map for accurate logo classification.

The design of LogoXpertNet is motivated by the observation that logo recognition differs fundamentally from generic object classification. While object-centric attention mechanisms focus on dominant semantic regions, logos frequently occupy small spatial regions and are characterized by repetitive textures, sharp edges, and high-frequency structures. The CLFF module addresses the limitation of single-layer recalibration by fusing early texture-rich features with deep semantic features before excitation, ensuring that discriminative low-level cues are preserved. The HSE-SAB module extends conventional spatial attention by incorporating hierarchical multi-scale pooling, enabling robust recognition under scale variation and partial occlusion. Finally, the FA-CBAM integrates frequency-domain information to explicitly enhance sensitivity to high-frequency logo patterns that are often suppressed by spatial-only attention mechanisms. Together, these components form a logo-specific attention framework rather than a generic feature enhancement pipeline.

#### Modified MobileNetv3 for feature extraction

In this research, the modified MobileNetV3 architecture plays a critical role in efficiently extracting discriminative features from images, which are essential for identifying and classifying logos. MobileNetV3 is a lightweight deep learning model designed for mobile and edge applications, leveraging depthwise separable convolutions to reduce the computational complexity while maintaining high performance. This efficiency is particularly beneficial in real-time image classification tasks, such as logo recognition, where both speed and accuracy are paramount.

The core of the logo classification system relies on the MobileNetV3 architecture, which is structured to capture hierarchical, multi-level features from input images. MobileNetV3 is modified by incorporating specialized blocks like the Bottleneck Block and CLFF Block to further enhance the model’s ability to extract fine-grained features, which are crucial for distinguishing between visually similar logos.

1. *Bottleneck Block*: The Bottleneck Block is a key component of MobileNetV3, designed to reduce computational load without sacrificing the quality of feature extraction. This block employs depthwise separable convolutions, which split the conventional convolution operation into two separate steps: a depthwise convolution and a pointwise convolution. The depthwise convolution applies a single filter to each input channel, while the pointwise convolution combines the results from different channels. This approach significantly reduces the number of parameters and computation, making MobileNetV3 particularly suitable for mobile devices or environments with limited computational resources. In logo classification, the Bottleneck Block helps in extracting essential high-level features from the image, such as shapes, textures, and edges, which are fundamental for distinguishing different logos.
2. *CLFF Block*: The CLFF (Channel-Layer Feature Fusion) Block is incorporated to capture more fine-grained features that are important for logo classification. Logos often exhibit subtle variations in size, color, and orientation, which require the model to focus on detailed visual cues. The CLFF Block aids in learning and fusing features from multiple channels and layers of the network, which helps the model capture the intricate patterns present in logos. This block allows MobileNetV3 to retain a rich set of features, ensuring the network is capable of accurately identifying logos even in the presence of noise or distortion.

MobileNetV3 architecture, with its efficient design based on depthwise separable convolutions and specialized blocks like the Bottleneck and CLFF Blocks, is particularly suited for logo classification. Its lightweight nature ensures that it can run on resource-constrained devices without compromising performance, making it an optimal choice for real-time logo recognition applications.

#### Cross-layer feature fusion (CLFF) module

**Fig. 2:**
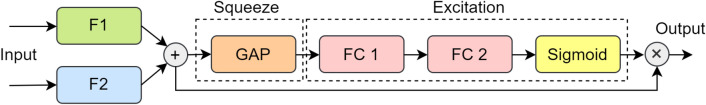
The architecture of the proposed CLFF module.

In this work, we propose the Cross-Layer Feature Fusion (CLFF) approach to enhance the SE module. By fusing features from both early and deep layers and applying the SE recalibration to the combined feature map, we aim to improve the model’s ability to recognize logos with varying levels of detail and complexity. Algorithm 1 shows the step-by-step process of the CLFF module, and Fig. 2 shows the architecture of the proposed CLFF module.

Let $F_1 \in \mathbb {R}^{C_1 \times H \times W}$ and $F_2 \in \mathbb {R}^{C_2 \times H \times W}$ represent the feature maps of the early and deep layers, respectively, where $C_1$ and $C_2$ are the number of channels and $H$ and $W$ are the spatial dimensions of the feature maps.

1. *Fusion of features*
  The feature maps $F_1$ and $F_2$ are fused using either concatenation or addition.
  If concatenation is used:
    1 $$\begin{aligned} F_{\text {fusion}} = \text {concat}(F_1, F_2) \end{aligned}$$
  Alternatively, if addition is used:
    2 $$\begin{aligned} F_{\text {fusion}} = F_1 + F_2 \end{aligned}$$
  This results in a fused feature map $F_{\text {fusion}}$ that combines the information from both layers.
2. *Squeeze operation*
  Global Average Pooling (GAP) is applied to the fused feature map $F_{\text {fusion}}$ to generate a global context vector $z$.
  The squeeze operation can be written as:
    3 $$\begin{aligned} z = \text {GAP}(F_{\text {fusion}}) \in \mathbb {R}^{(C_1 + C_2) \times 1 \times 1} \end{aligned}$$
  Here, $z$ is a $1 \times 1 \times (C_1 + C_2)$ vector that represents the global context of the fused feature map.
3. *Excitation operation*
  The squeezed vector $z$ is passed through two fully connected layers to capture channel dependencies.
  The excitation process is as follows:
    4 $$\begin{aligned} z_1 = W_1 z + b_1 \end{aligned}$$
    5 $$\begin{aligned} z_2 = W_2 z_1 + b_2 \end{aligned}$$
  Here, $W_1$ and $W_2$ are the weight matrices, and $b_1$ and $b_2$ are the bias terms.
  The output of the excitation process is passed through a sigmoid activation function to generate the attention weights $\textbf{a}$:
    6 $$\begin{aligned} \textbf{a} = \sigma (z_2) \end{aligned}$$
  Where $\sigma (\cdot )$ represents the sigmoid function.
4. *Recalibration of feature map*
  The attention weights $\textbf{a}$ are reshaped to match the dimensions of the fused feature map $F_{\text {fusion}}$ and are used to recalibrate the feature map.
  This can be written as:
    7 $$\begin{aligned} F_{\text {out}} = F_{\text {fusion}} \times \textbf{a} \end{aligned}$$
  Where $F_{\text {out}}$ is the output of the SE module with cross-layer feature fusion, which is a recalibrated feature map.

**Algorithm 1:**
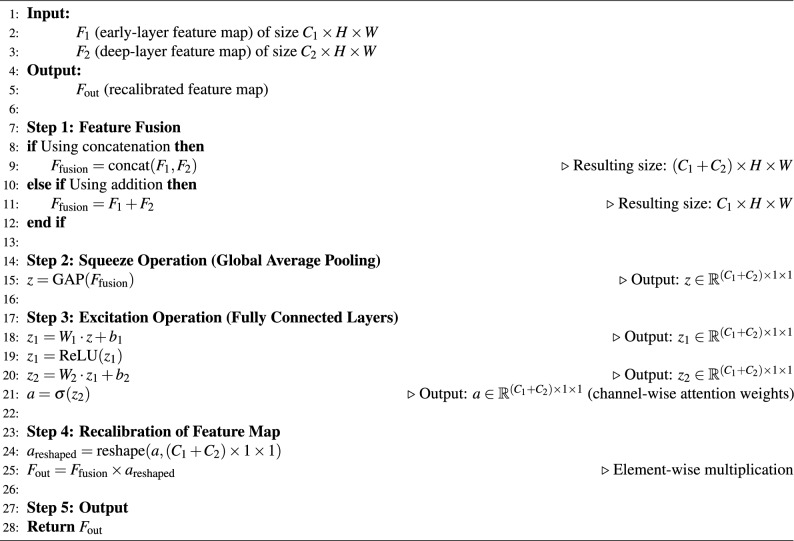
Cross-layer feature fusion (CLFF) module

#### Proposed hierarchical squeeze-excitation spatial attention block (HSE-SAB)

**Fig. 3:**
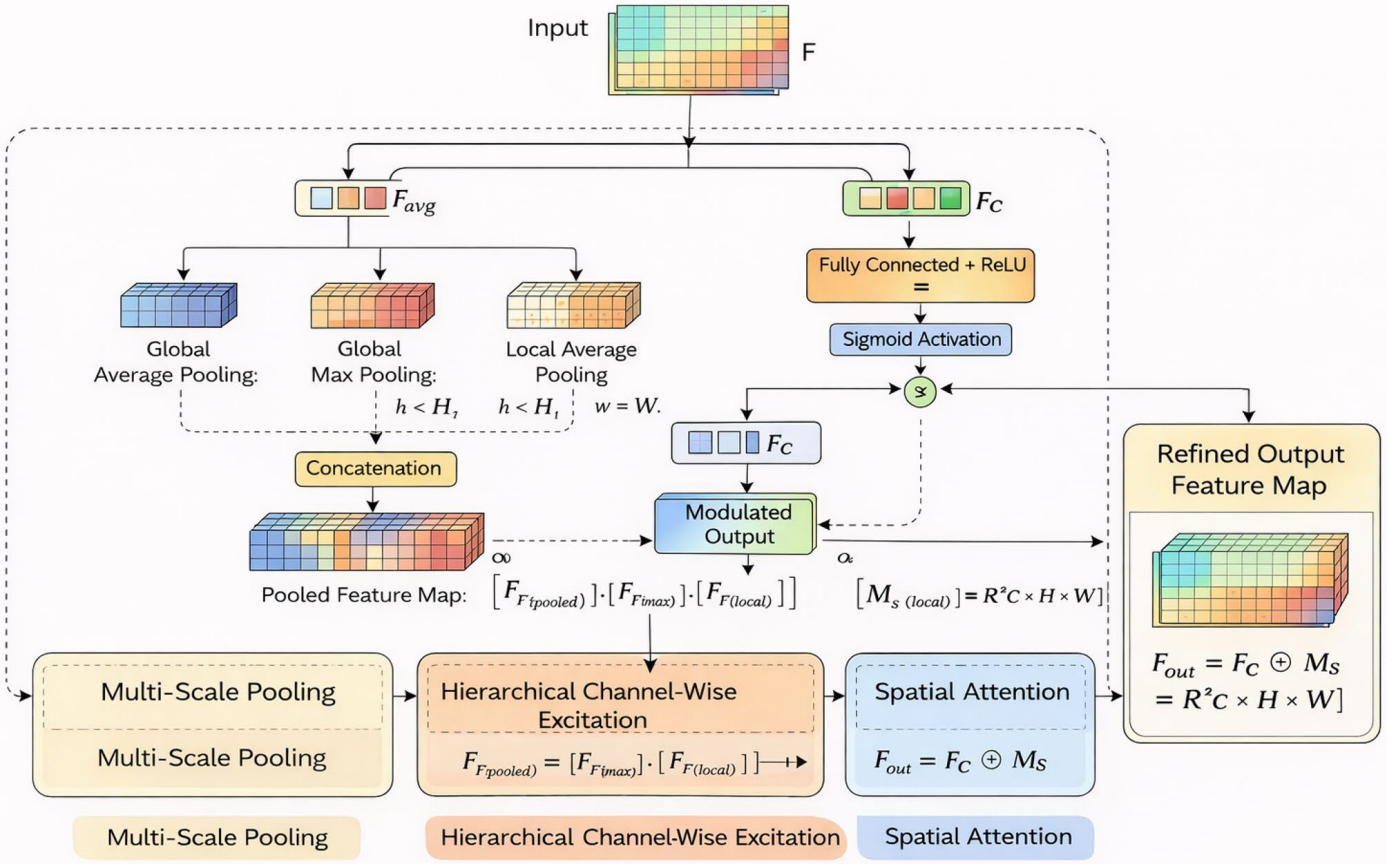
The architecture of the proposed HSE-SAB for logo classification.

In this work, we propose the Hierarchical Squeeze-Excitation Spatial Attention Block (HSE-SAB), a novel attention mechanism designed to enhance the discriminative power of deep convolutional networks for logo classification tasks. The HSE-SAB integrates multi-scale feature extraction, hierarchical channel-wise excitation, and spatial attention to dynamically focus on the most informative features within both the spatial and channel dimensions. This combination enables the model to effectively distinguish logos from diverse backgrounds, cope with variations in scale and appearance, and highlight the most relevant regions of interest in logo images. Figure 3 shows the architecture of the proposed HSE-SAB for logo classification.

The HSE-SAB operates in a multi-stage process, which consists of three core components: multi-scale pooling, channel-wise excitation, and spatial attention. Each of these stages contributes to improving the feature representation by capturing contextual information at different scales, emphasizing important features, and refining the spatial focus of the model.

1. *Multi-scale pooling*: The input feature map, denoted as $F \in \mathbb {R}^{C \times H \times W}$, where $C$, $H$, and $W$ represent the number of channels, height, and width, respectively, is first processed through multiple pooling operations to extract contextual information at various scales. The pooling operations capture both global and local context, which is particularly important for logo classification, where logos may appear at varying sizes and resolutions within the image. The multi-scale pooling is performed as follows:
  *Global average pooling (GAP)*: captures global contextual information by averaging the values across the spatial dimensions:
    8 $$\begin{aligned} F_{\text {avg}} = \text {GlobalAvgPool}(F) \in \mathbb {R}^{C \times 1 \times 1} \end{aligned}$$
  *Global max pooling (GMP)*: captures the most prominent feature across the entire spatial region:
    9 $$\begin{aligned} F_{\text {max}} = \text {GlobalMaxPool}(F) \in \mathbb {R}^{C \times 1 \times 1} \end{aligned}$$
  *Local average pooling (LAP)*: captures finer details by pooling over smaller spatial regions, allowing the model to focus on local structures such as edges and corners commonly found in logos:
    10 $$\begin{aligned} F_{\text {local}} = \text {LocalAvgPool}(F) \in \mathbb {R}^{C \times h \times w} \end{aligned}$$
    where $$h < H$$ and $$w < W$$.
  The pooled feature maps are concatenated to form a comprehensive representation of the input feature map:
  11 $$\begin{aligned} F_{\text {pooled}} = [F_{\text {avg}}; F_{\text {max}}; F_{\text {local}}] \in \mathbb {R}^{C \times 1 \times 1} \oplus \mathbb {R}^{C \times 1 \times 1} \oplus \mathbb {R}^{C \times h \times w} \end{aligned}$$
  where $\oplus$ denotes the concatenation operation.
2. *Hierarchical channel-wise excitation*: The next step involves channel-wise excitation, which allows the model to selectively emphasize informative channels and suppress less relevant ones. To achieve this, the concatenated pooled features $F_{\text {pooled}}$ are passed through two fully connected layers with ReLU activations, which model complex interchannel dependencies. This excitation mechanism enables the network to learn which channels are important for detecting logo patterns. The excitation process is as follows:
  12 $$\begin{aligned} F_{\text {excite}} = \text {ReLU}(W_2 \cdot \text {ReLU}(W_1 \cdot F_{\text {pooled}})) \end{aligned}$$
  where $W_1$ and $W_2$ are learnable weight matrices, and the ReLU activation introduces non-linearity to the model. The output of the excitation is then passed through a sigmoid activation function to generate channel-wise attention weights:
  13 $$\begin{aligned} F_{\text {c}} = F \odot \sigma (F_{\text {excite}}) \end{aligned}$$
  where $\odot$ represents element-wise multiplication, and $\sigma$ denotes the sigmoid function. This operation modulates the original feature map $F$ by highlighting the channels that contribute most to the classification of logos.
3. *Spatial attention*: To further enhance the model’s ability to detect logos, we introduce a spatial attention mechanism. This mechanism focuses the model’s attention on specific regions of the feature map that are highly relevant to logo classification. Spatial attention is particularly useful in identifying localized logo features, such as shapes and text, which are often spatially concentrated in certain regions of the image. The spatial attention map $M_s \in \mathbb {R}^{1 \times H \times W}$ is generated by applying a $1 \times 1$ convolution over the input feature map:
  14 $$\begin{aligned} M_s = \sigma (f^{1 \times 1}(F)) \end{aligned}$$
  where $f^{1 \times 1}$ denotes the convolution operation with a $1 \times 1$ kernel, and $\sigma$ is the sigmoid function that produces attention weights between 0 and 1. The spatial attention map is then applied to the channel-modulated feature map $F_{\text {c}}$, resulting in the final refined feature map $F_{\text {out}}$:
  15 $$\begin{aligned} F_{\text {out}} = F_{\text {c}} \odot M_s \end{aligned}$$
  This operation enhances the spatial saliency of logo-relevant regions by focusing on areas that contain key patterns and suppressing other less relevant areas.
4. *Final output*: The final output $F_{\text {out}}$ is a refined feature map that incorporates both channel-wise excitation and spatial attention. This dual-stage modulation process enables the model to adaptively focus on the most discriminative features for logo classification, improving its performance in identifying logos across varying backgrounds, scales, and appearances. The HSE-SAB combines these three mechanisms, multi-scale pooling, channel-wise excitation, and spatial attention into a unified block that enhances the network’s ability to detect logos with high accuracy. By capturing both global and local contextual information, emphasizing relevant channels, and focusing on important spatial regions, the HSE-SAB significantly improves logo classification performance, making it highly suitable for practical logo recognition applications.

Algorithm 2 gives the step-by-step process of the HSE-SAB block.

**Algorithm 2:**
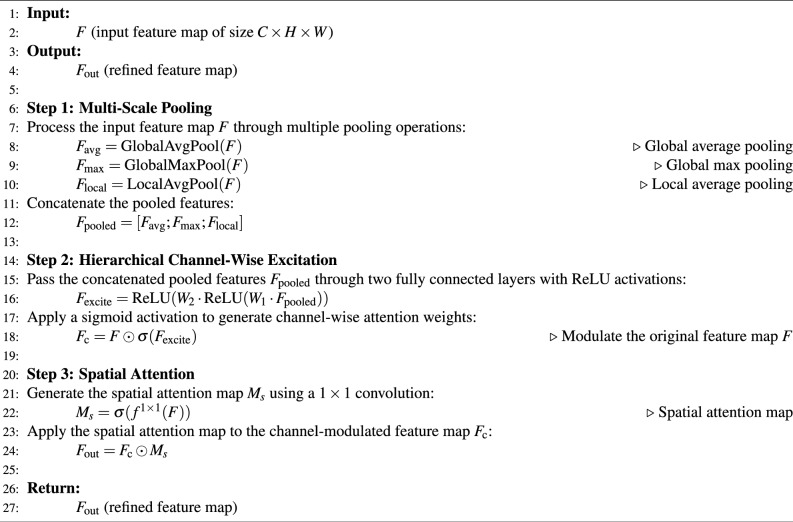
Hierarchical squeeze-excitation spatial attention block (HSE-SAB)

#### Proposed feature aware convolution block attention module

**Fig. 4:**
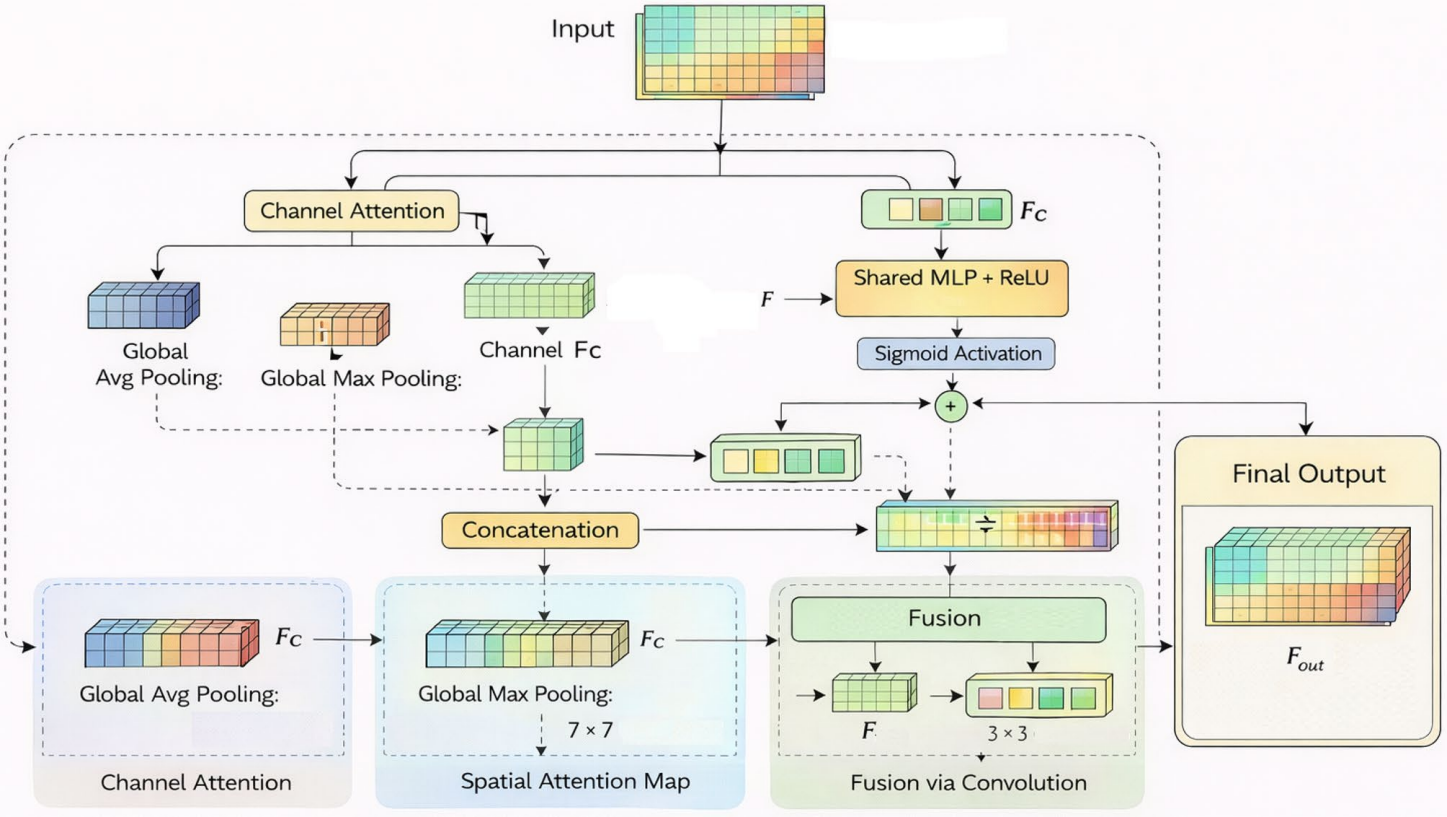
The architecture of the proposed FA-CBAM for logo classification.

In this paper, we propose the Feature-Aware Convolution Block Attention Module (FA-CBAM), a novel attention mechanism designed to enhance the ability of CNNs to focus on both spatial and frequency domain features that are essential for effective logo classification. Logos in real-world scenarios often exhibit complex patterns, obfuscation, scale variations, and background noise. To address these challenges, FA-CBAM integrates a frequency domain augmentation with the standard spatial attention mechanism, enabling the model to capture fine-grained, structure-aware, and frequency-specific features. This combination significantly improves the classification of logos, which may share common characteristics such as repeated shapes, gradients, and spatial structures. Figure 4 shows the architecture of the proposed FA-CBAM for logo classification.

The architecture of FA-CBAM consists of five main stages: channel attention, spatial attention, frequency domain augmentation, fusion via convolution, and final output refinement. Each of these components plays a critical role in enhancing the network’s ability to identify logos in diverse visual contexts.

1. *Channel attention*: The first component of FA-CBAM is the channel attention, which helps the network focus on the most relevant channels for logo recognition. This is achieved by aggregating spatial information using global average pooling and global max pooling, followed by a shared multi-layer perceptron (MLP) with ReLU activations. The channel attention map $M_c \in \mathbb {R}^{C \times 1 \times 1}$ is computed as follows:
  16 $$\begin{aligned} M_c(F) = \sigma (\text {MLP}(\text {AvgPool}(F)) + \text {MLP}(\text {MaxPool}(F))) \end{aligned}$$
  where $\sigma$ is the sigmoid activation function, ensuring the attention weights are within the range of 0 to 1. The output feature map $F_c$ is then obtained by modulating the original input feature map $F$ with the channel attention map:
  17 $$\begin{aligned} F_c = M_c(F) \odot F \end{aligned}$$
  Here, $\odot$ denotes the multiplication of elements in a channel, allowing the model to emphasize informative channels and suppress irrelevant ones based on the weights of channel attention.
2. *Spatial attention*: After channel-wise attention, the next component is spatial attention, which focuses on highlighting the regions of the feature map that are most relevant for detecting logos. The spatial attention map $M_s \in \mathbb {R}^{1 \times H \times W}$ is computed by first applying global average pooling and max pooling along the channel axis, followed by a 2D convolution. The spatial attention map is generated as follows:
  18 $$\begin{aligned} M_s(F_c) = \sigma (f^{7 \times 7}([\text {AvgPool}(F_c); \text {MaxPool}(F_c)])) \end{aligned}$$
  where $f^{7 \times 7}$ represents a $7 \times 7$ convolution, and the $[\cdot ; \cdot ]$ notation indicates the channel-wise concatenation of the pooled features. The sigmoid function $\sigma$ produces the spatial attention map, which allows the model to focus on the most relevant regions, such as the logo itself, while suppressing other irrelevant areas in the image.
3. *Frequency domain augmentation*: To incorporate frequency-domain features into the attention mechanism, we apply the Discrete Cosine Transform (DCT) to approximate the frequency content of the input feature map $F$. This is done by using the real part of the Fast Fourier Transform (FFT) as an approximation of the DCT:
  19 $$\begin{aligned} F_f = \text {DCT}(F) \approx \text {Re}(\text {FFT}(F)) \end{aligned}$$
  This transformation captures high-frequency components in the logo’s structure, such as fine-grained patterns, repetitive shapes, or embedded graphical features, which are often crucial for recognizing logos amidst complex backgrounds.
4. *Fusion via convolution*: The frequency domain features $F_f$ are then passed through a convolutional layer to extract more localized, frequency-aware features:
  20 $$\begin{aligned} F_f' = f^{\text {conv}}(F_f) \end{aligned}$$
  Next, the spatial attention map $M_s$ and the frequency-enhanced feature map $F_f'$ are concatenated to combine the spatial and frequency information. The concatenated features are refined using a $3 \times 3$ convolution, resulting in the final fused attention map $M_{sf}$:
  21 $$\begin{aligned} M_{sf} = \sigma (f^{3 \times 3}([M_s; F_f'])) \end{aligned}$$
  This operation combines the spatial and frequency information to generate a more refined attention map that highlights both spatially relevant regions and frequency-specific patterns within the image.
5. *Final output*: The final attention map $M_{sf}$ is applied to the channel-refined feature map $F_c$ to yield the final attention-enhanced output $F_{\text {out}}$:
  22 $$\begin{aligned} F_{\text {out}} = M_{sf} \odot F_c \end{aligned}$$
  This final output is a refined feature map that integrates both spatial and frequency-domain information, enhancing the model’s ability to detect logos by focusing on the most relevant features and regions in the image.

Algorithm 3 shows the step-by-step process for the FA-CBAM module. Table 4 shows the architectural comparison of different modules for logo classification.

**Algorithm 3:**
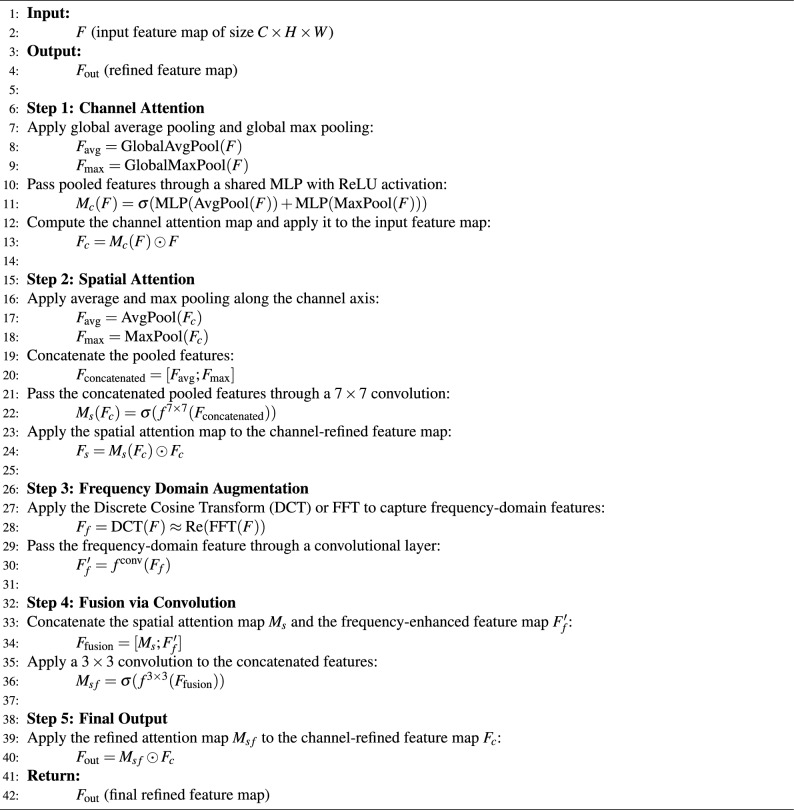
Feature-aware convolution block attention module (FA-CBAM)

**Table 4:** Architectural novelty comparison: LogoXpertNet versus SE block versus CBAM.

Aspect	SE	CBAM	LogoXpertNet Components	Novelty in LogoXpertNet
Core Idea	Channel-wise attention	Sequential channel + spatial attention	Multi-stage fusion of channel, spatial, and frequency attention	Integrates frequency-domain information (DCT) alongside spatial-channel attention
Attention Type	Channel	Channel → Spatial (sequential)	Hierarchical + Cross-Layer + Frequency-Aware	Combines CLFF for cross-layer fusion, HSE-SAB for multi-scale pooling, and FA-CBAM for frequency enhancement
Spatial Attention	✗ No spatial attention	✓ Uses avg+max pooling + conv (7× 7)	✓ Enhanced via multi-scale pooling (avg, max, min) + local pooling	Hierarchical pooling captures global + local context; spatial attention refined with frequency features
Channel Attention	✓ Global avg pooling + FC layers	✓ Global avg + max pooling + shared MLP	✓ CLFF fuses early + deep layer features before excitation	Cross-layer feature fusion before squeeze-excitation improves multi-level feature integration
Frequency Domain	✗ Not considered	✗ Not considered	✓ FA-CBAM uses DCT/FFT for frequency-domain augmentation	First to integrate frequency cues for logo structure (edges, textures, repetitive patterns)
Multi-Scale Processing	✗ Single-scale pooling	✗ Single-scale pooling	✓ HSE-SAB uses avg, max, min, and local average pooling	Captures fine-grained local details crucial for small/occluded logos
Feature Fusion	✗ No cross-layer fusion	✗ No cross-layer fusion	✓ CLFF concatenates/adds early + deep layer features	Improves handling of varying logo complexities and scales
Application Focus	General-purpose CNN enhancement	General object detection/classification	Logo-specific optimization	Designed for high intra-class variation, stylized text, background clutter
Lightweight Design	✓ Lightweight	✓ Moderate overhead	✓ Built on MobileNetV3 with efficient blocks	Maintains efficiency while adding multi-modal attention
Key Innovation	Channel recalibration	Sequential spatial-channel attention	Unified spatial-channel-frequency attention with cross-layer fusion	First logo classification model to combine spatial, channel, and frequency attention in a lightweight architecture

Unlike generic object recognition, logo classification presents unique challenges, including fine-grained inter-class similarity, stylized typography, repetitive geometric structures, and frequent scale variation. These characteristics motivate the use of specialized feature fusion and attention mechanisms. The CLFF module preserves low-level texture information while integrating high-level semantic features, enabling discrimination between visually similar logos. The HSE-SAB introduces hierarchical multi-scale attention to improve robustness to scale changes and partial occlusions. The FA-CBAM further enhances logo recognition by incorporating frequency-domain information, which is particularly effective for capturing high-frequency logo patterns such as edges, contours, and repetitive structures. Together, these modules form a logo-specific feature enhancement strategy rather than a generic attention pipeline.

## Experimental results and analysis

### Dataset definition

#### FlickrLogos-32

**Fig. 5:**
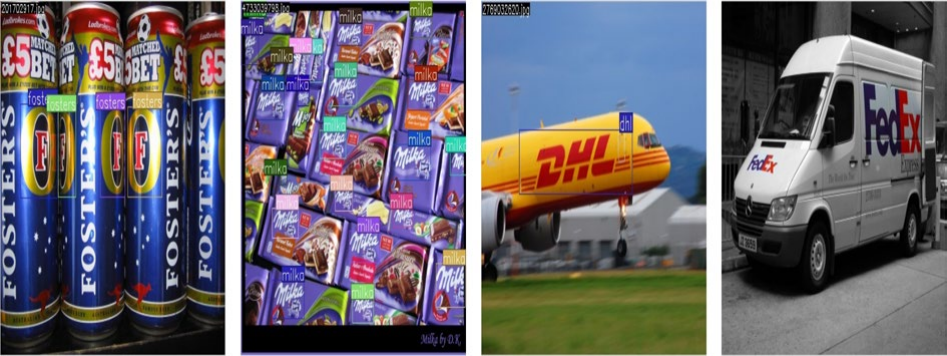
The sample images of Flickr32-Logos dataset.

FlickrLogos-32^28^ is one of the most well-known datasets for logo classification tasks. It contains 32 logo categories, with around 8,000 images in total. The images in this dataset are sourced from the Flickr platform, making it suitable for evaluating models in a semi-controlled environment, with varying backgrounds, resolutions, and lighting conditions. The logos are often embedded in real-world scenes, providing an excellent challenge for classification tasks. This dataset is widely used in academic research and benchmarking, making it a go-to resource for small to medium-scale logo recognition tasks. It offers a balance between dataset size and complexity, making it ideal for training deep learning models without overwhelming them with excessive data. Figure 5 shows the sample images of the Flickr32-Logos dataset.

#### BelgaLogos

**Fig. 6:**
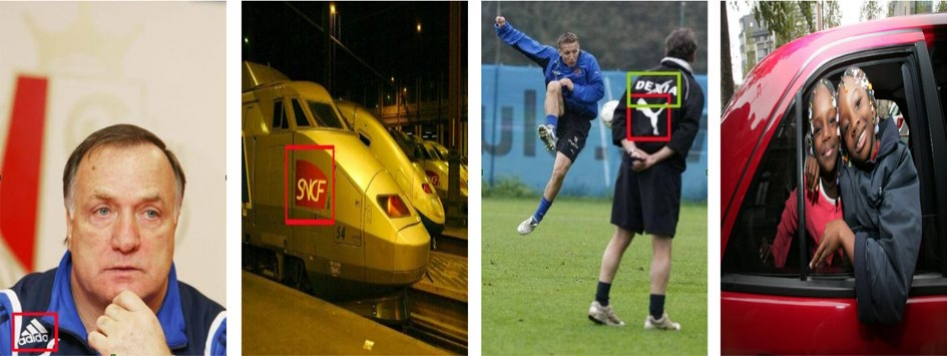
The sample images of the BelgaLogos dataset.

BelgaLogos^29^ is a relatively small dataset specifically curated for Belgian logos. It contains around 3000 images spread across 15 different logo categories, making it an ideal dataset for focused logo classification tasks, particularly in regional or industry-specific settings. The images are captured in real-world environments, offering variability in terms of background, orientation, and scale. This dataset provides a good balance between size and complexity, offering enough examples to train models while not being too large to make experimentation too costly. BelgaLogos is especially useful if you are targeting logo classification in European or regional contexts. Figure 6 shows the sample images of the BelgaLogos dataset.

#### WebLogo-2M

**Fig. 7:**
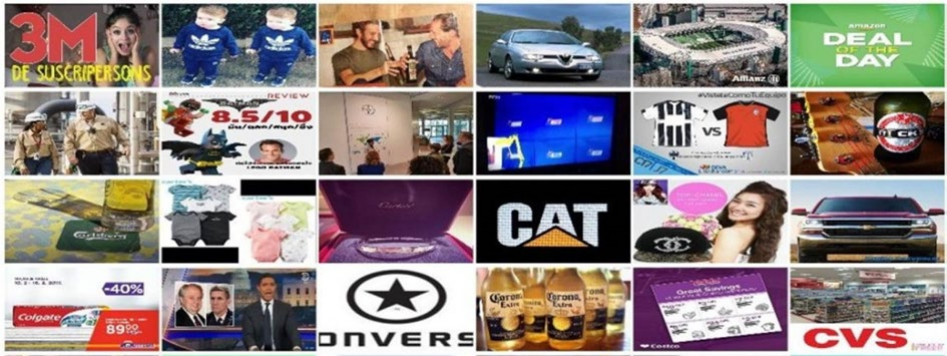
The sample images of the WebLogo-2M dataset.

The WebLogo-2M dataset consists of approximately 2.19 million real-world images spanning 194 logo categories, collected from diverse web sources and characterized by significant label noise, scale variation, occlusion, and background clutter. In this work, we use the full WebLogo-2M dataset without class or image filtering. The dataset is split into 1,752,000 images for training, 219,000 images for validation, and 219,000 images for testing. All splits preserve the original class distribution to ensure balanced evaluation. Figure 7 shows the sample images of the WebLogo-2M dataset.

#### Dataset split protocol and evaluation scope

To improve the transparency and interpretability of the reported results, we clarify the data partitioning protocol used in this study. For each dataset, the train, validation, and test sets were constructed at the image level using class-balanced splitting so that the class distribution remained consistent across partitions. For WebLogo-2M, the dataset was divided into 1,752,000 images for training, 219,000 images for validation, and 219,000 images for testing, with the original class distribution preserved. The reported results therefore reflect performance on the specific benchmark splits used in this work.

We emphasize that benchmark performance can be influenced by the difficulty of the dataset, the degree of inter-class separability, the presence of visually repetitive patterns, and the exact split construction. Accordingly, the near-ceiling accuracy values reported in this study should be interpreted with caution and in the context of the evaluated benchmark setup rather than as unconditional evidence of solved real-world logo classification.

### Evaluation metrics

Evaluation metrics are essential tools for assessing the performance of deep learning models. In this section, we present several common metrics that are widely used in classification tasks. Unless otherwise stated, the primary metric reported in this study is top-1 image-level classification accuracy computed on the held-out test set. Precision, recall, and F1-score are reported to provide complementary views of multiclass performance, and AUC is included as an additional summary indicator of class discrimination. Because different studies may report results under different metric definitions, dataset partitions, or evaluation protocols, direct numerical comparisons should be interpreted carefully, especially when the reported performance approaches the ceiling of a benchmark.

#### Accuracy

Accuracy is the ratio of the number of correct predictions to the total number of predictions. It provides a general measure of how well the model is performing overall.

23 $$\begin{aligned} \text {Accuracy} = \frac{TP + TN}{TP + TN + FP + FN} \end{aligned}$$

Where $TP$ is True Positive, $TN$ is True Negative, $FP$ is False Positive, and $FN$ is the False Negatives.

#### Precision

Precision measures the proportion of true positive predictions out of all positive predictions made by the model. It is an important metric when the cost of false positives is high.

24 $$\begin{aligned} \text {Precision} = \frac{TP}{TP + FP} \end{aligned}$$

Where $TP$ is True Positive, and $FP$ is False Positive.

#### Recall

Recall (also known as Sensitivity or True Positive Rate) measures the proportion of actual positive instances that were correctly identified by the model. It is important when the cost of false negatives is high.

25 $$\begin{aligned} \text {Recall} = \frac{TP}{TP + FN} \end{aligned}$$

Where $TP$ is True Positive, and $FN$ is False Negative.

#### F1 score

The F1 Score is the harmonic mean of precision and recall. It balances the two metrics by giving a single score that considers both false positives and false negatives, making it a useful metric when the class distribution is imbalanced.

26 $$\begin{aligned} \text {F1 Score} = 2 \cdot \frac{\text {Precision} \cdot \text {Recall}}{\text {Precision} + \text {Recall}} \end{aligned}$$

#### Area under the curve (AUC)

The Area Under the Curve (AUC) is a performance measurement for classification problems at various threshold settings. It represents the likelihood that the model will rank a randomly chosen positive instance higher than a randomly chosen negative instance.

27 $$\begin{aligned} \text {AUC} = \int _{0}^{1} \text {TPR}(\text {FPR}) \, d\text {FPR} \end{aligned}$$

Where $\text {TPR}$ is the true positive rate and $\text {FPR}$ is the false positive rate, defined as $\frac{FP}{FP + TN}$.

We compute AUC using OvR ROC curves and report macro-AUC over all classes.

### Model training parameters

The datasets used were divided based on their predefined splits, with 80% allocated to training, 10% to validation, and 10% to testing. To prevent data leakage, dataset splitting was performed prior to data augmentation, ensuring that no exact or near-duplicate images appeared across the training, validation, and test sets. We conducted duplicate and near-duplicate detection. First, we removed exact or visually identical duplicates using perceptual hashing: images with identical perceptual hashes (Hamming distance $\le$ 0) were treated as duplicates. Second, to capture near-duplicates caused by cropping, resizing, watermark overlays, and mild photometric edits, we extracted fixed image embeddings using CLIP ViT-B/32 and computed cosine similarities. Two images were considered near-duplicates when the cosine similarity was $\ge$ 0.985. For each duplicate/near-duplicate group, we retained one representative image and removed the remaining images. After this cleaning step, we performed the train/validation/test split, and augmentation was applied only to the training set.

Using this protocol, we detected 1842 duplicate pairs and 3,917 near-duplicate pairs. Under our adopted policy of keeping one representative image per group, we removed 1289 images in total and retained 199,784 images for subsequent splitting and training. Because grouping was performed prior to splitting, 0 duplicate/near-duplicate groups co-occur across train/val/test, i.e., the number of prevented cross-split co-occurrences equals the number of multi-image groups and the number of blocked cross-split pairs equals the number of duplicate/near-duplicate pairs within those groups.

As an explicit verification against accidental duplication or leakage, we computed CLIP ViT-B/32 embeddings for all images and, for each test image, measured the maximum cosine similarity to any training image. We report the distribution and maximum similarity. Under our duplicate-control threshold (cosine $\ge$ 0.985), no test image has a training near-duplicate above the threshold, confirming that the reported performance is not attributable to duplicate co-occurrence across splits.

All augmented samples were generated exclusively within their respective splits. Since logo classification is a multi-class problem where brand identity defines the class label, class-level separation is inherently preserved. Fixed random seeds were used to ensure reproducibility of the data splits. This ensured that the model’s evaluation was based on truly unseen data. The model was trained for 150 epochs with an initial learning rate of 0.001, using the Adam optimizer and L2 regularization (coefficient of 0.0005). To prevent overfitting, learning rate decay was applied every 10 epochs, and the training process was evaluated with five different random seeds (0, 1, 42, 123, 1234) to confirm the model’s stability and reproducibility. Before feeding the dataset to model, they underwent denoising using a Gaussian filter to remove noise and improve image quality. Normalization was applied to each image by subtracting the mean pixel value and dividing by the standard deviation, using the dataset-specific values for efficient training. To increase the model’s robustness, data augmentation techniques were employed, including random rotations, horizontal flipping, and brightness adjustments, each with a probability of 0.5, ensuring diverse representations of the logos during training.

All experiments were implemented using PyTorch (v2.3.0) with CUDA 11.8. Training and evaluation were conducted on a workstation equipped with an NVIDIA RTX 3090 GPU (24 GB VRAM) and an Intel Core i9 CPU, with 64 GB system memory. The model was trained using a batch size of 64 and an input resolution of 224 × 224 pixels. All experiments were performed using single-precision floating point (FP32).

The total training time for WebLogo-2M was approximately 35 hours for 150 epochs. Inference speed (FPS) was measured using batch size 1 with fixed input resolution, after a warm-up phase of 100 iterations to stabilize runtime performance. FPS measurements report pure model inference time and exclude data loading and preprocessing overhead to ensure fair comparison with prior work. CPU inference was evaluated using a single-threaded setup.

**Fig. 8:**
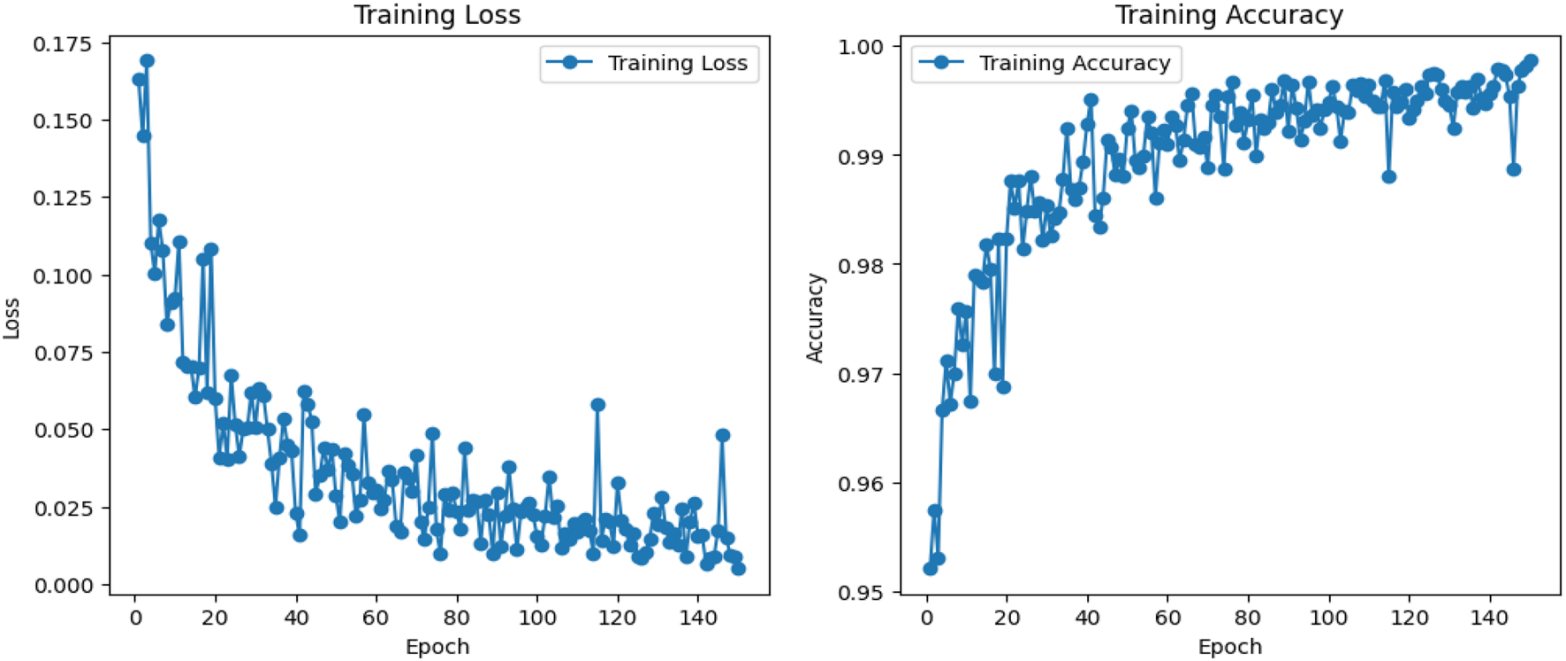
The graph of the training accuracy and training loss.

Figure 8 shows the training accuracy and training loss.

No manual filtering, relabeling, or label-noise correction was applied to WebLogo-2M. All images were used in their original form as provided by the dataset authors. This choice ensures that the reported results reflect performance under realistic, noisy web-scale conditions rather than curated or cleaned subsets.

### Comparison of the proposed model with baseline models

Table 5 compares various logo recognition methods across three datasets, FlickrLogos-32, BelgaLogos, and WebLogo-2M, using consistent hyperparameters to ensure fair evaluation. On FlickrLogos-32, prior methods such as Fujitake et al. achieve strong performance with an accuracy of 92.35% and an F1 score of 95.43%. However, the proposed method significantly outperforms all competitors, reaching an accuracy of 99.89% along with near-perfect precision, recall, F1 score, and AUC. Similarly, on the BelgaLogos dataset, existing models like Suren et al. achieve around 92.75% accuracy, while the proposed model again excels with an accuracy of 99.83% and similarly high auxiliary metrics.

The WebLogo-2M dataset follows a similar pattern: top-performing prior methods, such as those by Kumar et al., achieve approximately 91.94% accuracy, whereas the proposed method achieves a remarkable 99.92% accuracy, along with precision, recall, F1 score, and AUC all exceeding 99.9%. Across all three datasets, the proposed approach consistently demonstrates substantial improvements over state-of-the-art methods, affirming its robustness and superior performance in logo recognition tasks.

**Table 5:** Comparison of the Proposed Model with Baseline Models.

Dataset	Reference	Accuracy (%)	Precision (%)	Recall (%)	macro-F1 (%)	AUC (%)
FlickrLogos-32	Fujitake et al.^5^	92.35 ± 0.11	94.61 ± 0.05	94.52 ± 0.31	95.43 ± 0.22	94.82 ± 0.11
	Hou et al.^30^	91.85 ± 0.15	93.11 ± 0.20	92.34 ± 0.22	92.72 ± 0.17	93.89 ± 0.19
	Sharma et al.^31^	94.01 ± 0.12	94.43 ± 0.14	93.72 ± 0.18	94.08 ± 0.13	94.95 ± 0.20
	Sahel et al.^32^	93.57 ± 0.09	94.12 ± 0.17	93.12 ± 0.10	93.55 ± 0.11	93.95 ± 0.18
	Ma et al.^33^	95.67 ± 0.04	98.58 ± 0.05	99.06 ± 0.08	97.82 ± 0.12	98.61 ± 0.07
	LogoXpertNet	**99.89 ± 0.02**	**99.96 ± 0.03**	**99.92 ± 0.01**	**99.93 ± 0.04**	**99.98 ± 0.02**
BelgaLogos	Suren et al.^34^	92.75 ± 0.22	94.30 ± 0.16	93.11 ± 0.17	93.68 ± 0.19	93.84 ± 0.12
	Kandula et al.^35^	94.12 ± 0.15	94.75 ± 0.13	94.23 ± 0.14	94.49 ± 0.16	94.87 ± 0.17
	Bao et al.^36^	94.85 ± 0.10	95.05 ± 0.12	94.33 ± 0.14	94.69 ± 0.11	95.15 ± 0.14
	Chu et al.^23^	93.98 ± 0.14	94.32 ± 0.15	93.67 ± 0.13	94.07 ± 0.14	94.46 ± 0.12
	Ma et al.^33^	92.48 ± 0.06	94.36 ± 0.08	97.11 ± 0.12	94.16 ± 0.09	95.01 ± 0.12
	LogoXpertNet	**99.83 ± 0.03**	**99.94 ± 0.02**	**99.93 ± 0.01**	**99.92 ± 0.02**	**99.94 ± 0.01**
WebLogo-2M	Kumar et al.^37^	91.94 ± 0.24	92.34 ± 0.29	91.11 ± 0.31	91.71 ± 0.22	92.12 ± 0.25
	Fehrvri et al.^38^	92.65 ± 0.20	92.90 ± 0.28	92.16 ± 0.32	92.53 ± 0.23	92.99 ± 0.20
	Wang et al.^24^	93.11 ± 0.18	93.42 ± 0.19	92.73 ± 0.22	93.07 ± 0.21	93.42 ± 0.18
	Su et al.^25^	94.21 ± 0.22	94.51 ± 0.23	93.82 ± 0.24	94.16 ± 0.22	94.50 ± 0.21
	Ma et al.^33^	89.46 ± 0.13	92.36 ± 0.08	93.16 ± 0.14	94.02 ± 0.08	93.24 ± 0.08
	LogoXpertNet	**99.92 ± 0.02**	**99.97 ± 0.06**	**99.94 ± 0.01**	**99.92 ± 0.02**	**99.95 ± 0.03**

Significant values are in bold.

**Fig. 9:**
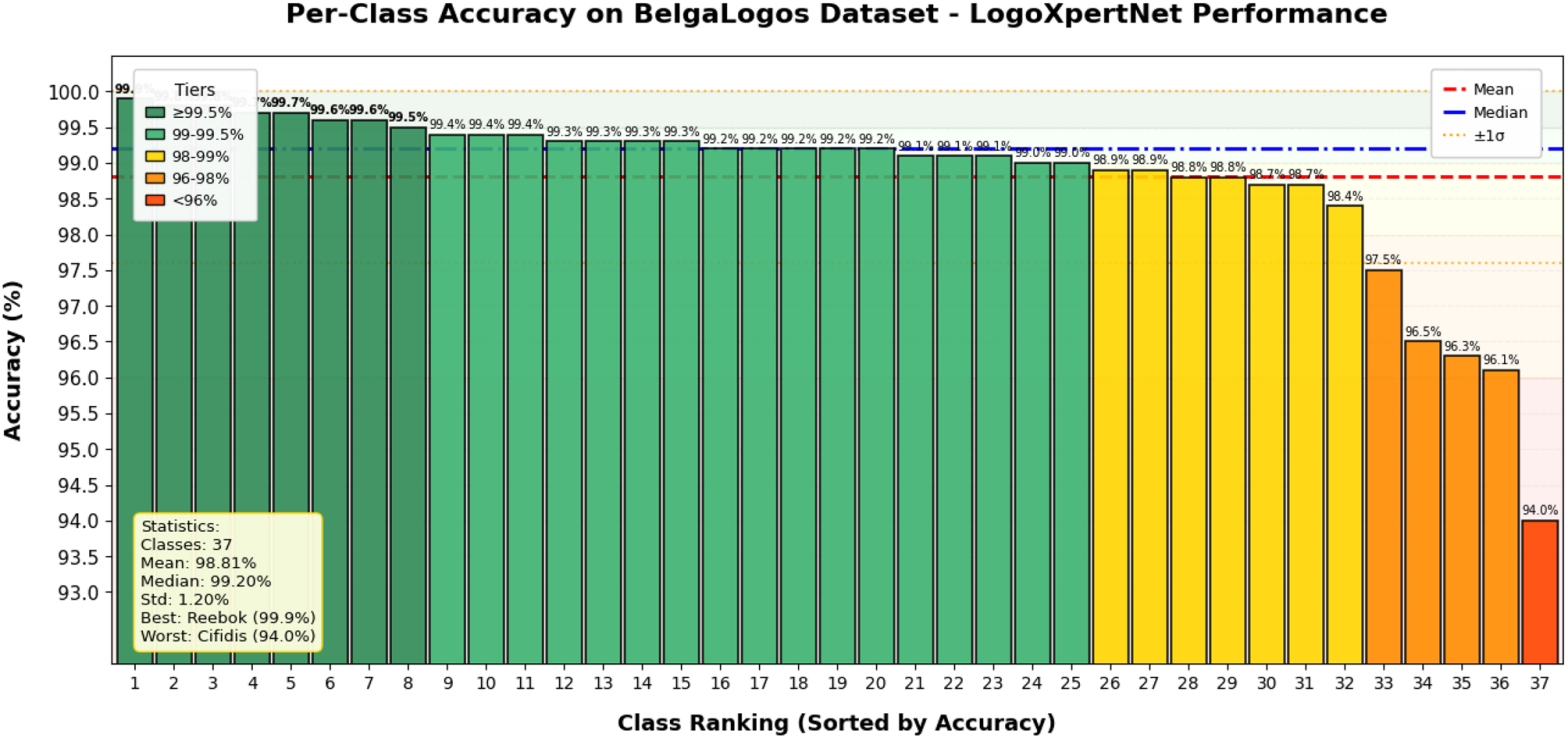
The per-class accuracy of the BelgaLogos dataset.

**Fig. 10:**
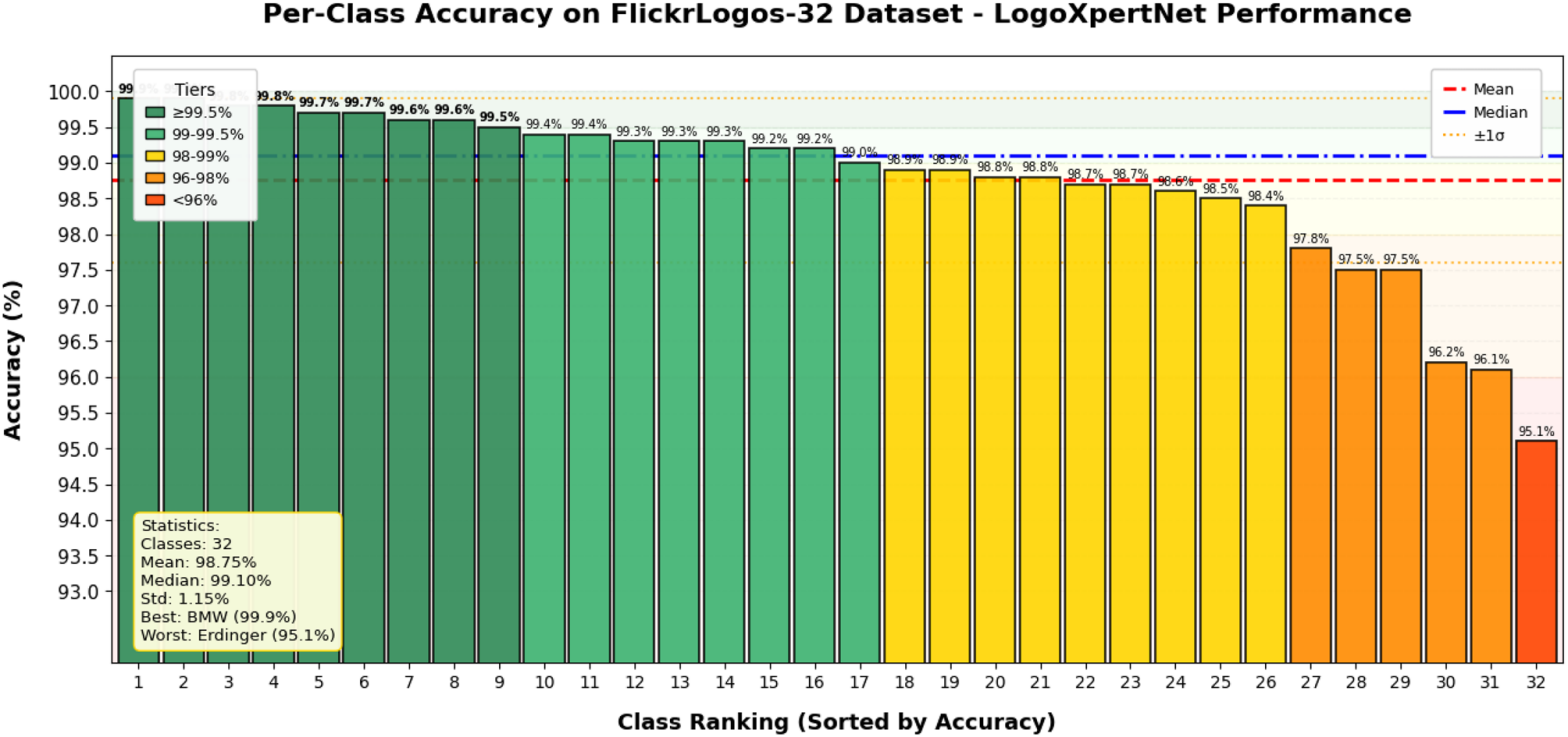
The per class accuracy of FlickrLogos-32 dataset.

**Fig. 11:**
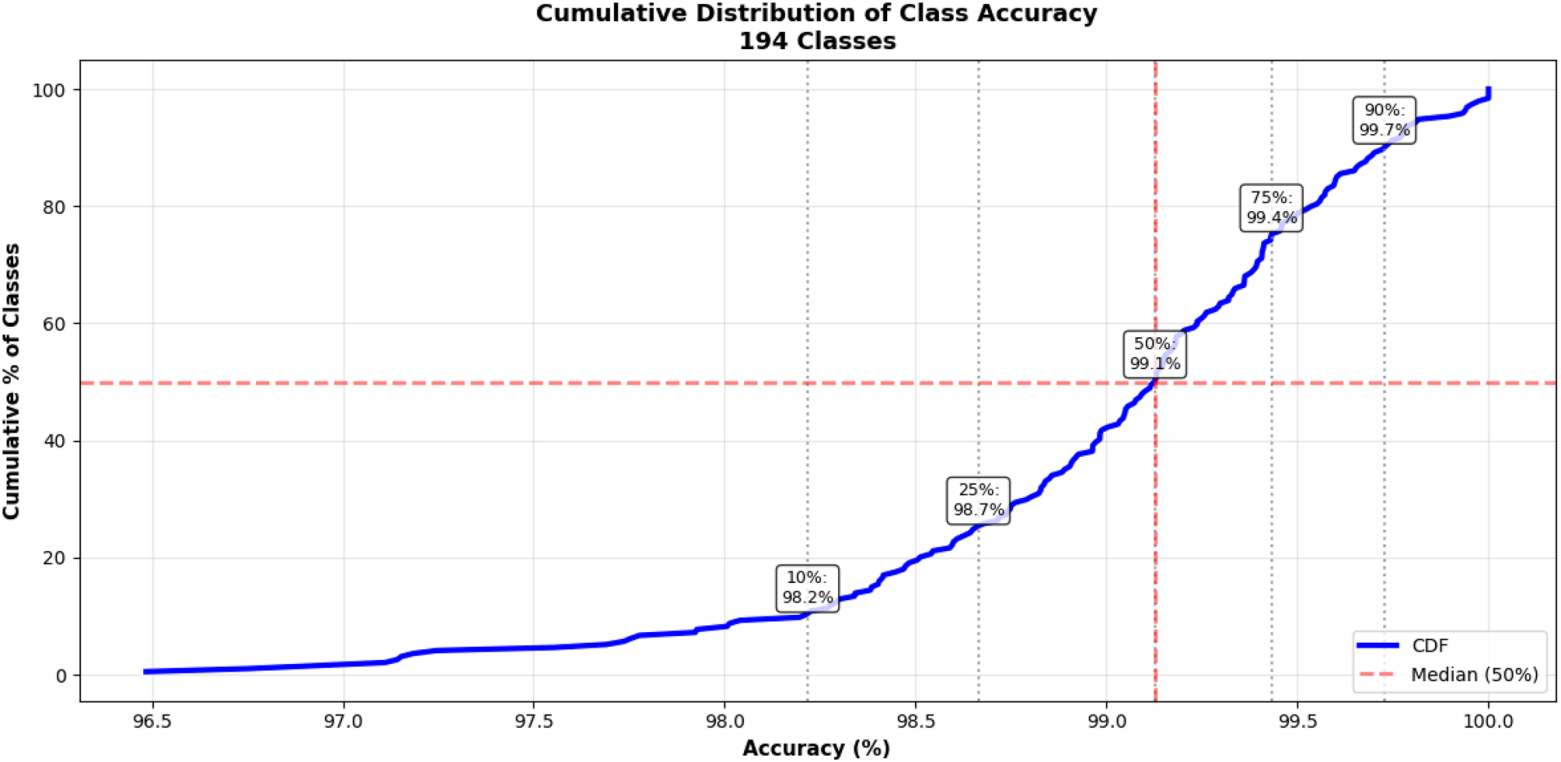
The per class accuracy of the WebLogo-2M dataset.

The per-class results of the proposed model for each dataset are shown in Fig. 9 for BelgaLogos dataset, Fig. 10 for FlickrLogos-32 dataset, and Fig. 11 for WebLogos-2M dataset, respectively. There are 194 classes, due to which it’s difficult to add a bar graph for each class. Therefore, we added a line graph to show the accuracy.

### Comparison of the proposed LogoXpertNet with different seed values

Table 6 compares the performance of LogoXpertNet across five different random seeds (0, 1, 42, 123, 1234) on three logo classification datasets, FlickrLogos-32, BelgaLogos, and WebLogo-2M, evaluated using accuracy, precision, recall, F1-score, and AUC. Performance varies slightly by seed, with seed 42 consistently yielding the best results across all datasets, achieving the highest accuracy (99.31% on FlickrLogos-32, 99.83% on BelgaLogos, and 99.92% on WebLogo-2M) and AUC (up to 99.68%, 99.94%, and 99.95%, respectively). BelgaLogos and WebLogo-2M show higher overall performance and lower variability, while FlickrLogos-32 exhibits slightly lower metrics and greater seed-dependent fluctuations, particularly in precision. The small standard deviations across all seeds confirm the model’s robustness to random initialization, maintaining consistently high performance regardless of the chosen seed.

**Table 6:** Comparison of LogoXpertNet with 5 different seed values.

Dataset	Seed	Accuracy (%)	Precision (%)	Recall (%)	macro-F1 (%)	AUC (%)
FlickrLogos-32	0	99.23 ± 0.1	98.54 ± 0.6	98.41 ± 0.1	98.29 ± 0.1	99.57 ± 0.1
	1	99.14 ± 0.4	98.67 ± 0.3	98.39 ± 0.2	98.18 ± 0.2	99.47 ± 0.2
	42	99.31 ± 0.3	98.74 ± 0.2	98.53 ± 0.3	98.32 ± 0.4	99.68 ± 0.1
	123	99.21 ± 0.1	98.65 ± 0.1	98.47 ± 0.1	98.23 ± 0.3	99.58 ± 0.1
	1234	99.18 ± 0.2	98.57 ± 0.2	98.26 ± 0.2	98.47 ± 0.2	99.43 ± 0.2
BelgaLogos	0	99.80 ± 0.04	99.92 ± 0.03	99.91 ± 0.02	99.90 ± 0.03	99.92 ± 0.03
	1	99.82 ± 0.03	99.93 ± 0.02	99.92 ± 0.03	99.91 ± 0.02	99.93 ± 0.02
	42	99.83 ± 0.03	99.94 ± 0.02	99.93 ± 0.01	99.92 ± 0.02	99.94 ± 0.01
	123	99.81 ± 0.04	99.91 ± 0.04	99.90 ± 0.03	99.90 ± 0.04	99.92 ± 0.03
	1234	99.82 ± 0.03	99.93 ± 0.03	99.92 ± 0.02	99.91 ± 0.03	99.93 ± 0.02
WebLogo-2M	0	99.89 ± 0.03	99.95 ± 0.07	99.92 ± 0.02	99.90 ± 0.03	99.93 ± 0.04
	1	99.90 ± 0.02	99.96 ± 0.05	99.93 ± 0.03	99.91 ± 0.02	99.94 ± 0.03
	42	99.92 ± 0.02	99.97 ± 0.06	99.94 ± 0.01	99.92 ± 0.02	99.95 ± 0.03
	123	99.91 ± 0.03	99.96 ± 0.05	99.93 ± 0.02	99.91 ± 0.03	99.94 ± 0.03
	1234	99.90 ± 0.02	99.95 ± 0.06	99.92 ± 0.02	99.90 ± 0.03	99.93 ± 0.04

This research assumes that publicly available datasets are sufficiently representative of real-world logo recognition scenarios, that the proposed attention mechanisms generalize well across diverse and noisy environments, and that the model’s lightweight design based on MobileNetV3 is suitable for real-time deployment on resource-constrained devices. The limitations include dependence on the quality and variety of training data, potential challenges with highly distorted, occluded, or stylized logos not well-covered in the datasets, and the computational and resource demands of the proposed modules despite the lightweight backbone, which may still hinder deployment on very low-power devices.

Although LogoXpertNet achieves very strong performance on the evaluated benchmark datasets, several limitations should be acknowledged. First, the model remains less reliable for very small, low-resolution, heavily occluded, distorted, or noisy logos, partly because such cases are inherently difficult and may be under-represented in the available training data. Second, near-ceiling benchmark accuracy should be interpreted cautiously, as it may partially reflect the characteristics of the evaluated datasets, including class composition, class separability, repeated visual motifs, and the exact train/validation/test split protocol. Third, strong performance on benchmark classification tasks does not necessarily imply equivalent robustness in open-world deployment settings involving unseen brands, domain shift, severe compression, motion blur, or highly cluttered scenes. Future work will therefore focus on more challenging cross-domain evaluation, harder real-world test conditions, and additional strategies for small-logo and occlusion-robust recognition.

### Comparison of the proposed model with lightweight models

The comparative analysis indicates that LogoXpertNet performs strongly relative to the lightweight baselines evaluated under the same experimental pipeline. On the benchmark splits used in this study, LogoXpertNet achieved very high accuracy on FlickrLogos-32 while maintaining a modest parameter count and competitive computational cost. However, because the observed values are close to the performance ceiling of the benchmark, they should be interpreted cautiously. These results likely reflect both the effectiveness of the proposed architecture and the characteristics of the evaluated datasets, including class separability, benchmark difficulty, and split construction. Therefore, we avoid claiming that the observed benchmark gains automatically translate to all real-world logo-classification settings.

We report inference speed in frames-per-second (FPS) excluding disk I/O, data loading, and CPU-side preprocessing, and we state this explicitly to avoid ambiguity. All FPS results in this paper (including baselines) are measured by us under the same controlled settings: identical hardware (GPU/CPU), identical deep learning framework implementation, identical input resolution, identical precision mode, and identical batch size. Timing is obtained after a warm-up phase and computed from repeated forward passes with device synchronization to reflect true inference time on the accelerator (Table 7).

**Table 7:** Comparative analysis: LogoXpertNet vs. lightweight models.

Model	Architecture	Params (M)	GFLOPs	Accuracy (%)	FPS (CPU)	FPS (GPU)
MobileNetV3	Mobile-optimized CNN, SE blocks	5.4	0.42	89.51	122	310
EfficientNet-Lite	Compound scaling, MBConv	10.8	0.34	92	95	280
ShuffleNetV2	Channel shuffle, lightweight CNN	**4.9**	**0.28**	~ 88	151	400
LogoXpertNet	MobileNetV3 + CLFF + HSE-SAB + FA-CBAM	6.1	0.35	**99.89**	**90**	**220**

FPS values are reported for batch size 1 at 224 × 224 input resolution, excluding data loading overhead, after a warm-up phase.

Significant values are in bold.

GFLOPs estimates the theoretical number of arithmetic operations for a forward pass, but practical FPS also depends on memory bandwidth and access patterns, kernel/operator efficiency, framework-level optimizations, parallelism, and device utilization. Consequently, FPS does not necessarily scale linearly with GFLOPs, and models with similar GFLOPs can show different FPS even on the same hardware.

### Error and confusion analysis

The analysis of misclassification patterns in Table 8 reveals that logo confusion is primarily driven by visual similarities in color schemes, iconographic elements, and industry-specific design conventions. The most frequent confusion pairs demonstrate that color palette overlap serves as the primary source of misclassification, as evidenced by the high confusion rates between Coca-Cola and Pepsi (both featuring red/blue combinations), Texaco and Shell (sharing red/yellow petroleum branding), and Stella Artois with Carlsberg (employing similar gold/red/white beer crest aesthetics). Shape-based similarities also contribute significantly to misclassifications, particularly with animal iconography such as the horse logos of Ferrari and Ford, as well as geometric patterns like the Bavarian blue/white diamonds shared between Paulaner and Erdinger. Additionally, industry context creates predictable confusion clusters, with logistics companies (FedEx/DHL/UPS), automotive manufacturers (BMW/Ford), and beverage brands frequently being misclassified within their respective sectors, suggesting that the model relies heavily on domain-specific visual cues that may be insufficient for fine-grained discrimination among visually similar competitors.

**Table 8:** Top-10 confusion pairs (most frequent misclassifications).

Rank	True class	Predicted As	Frequency	Visual similarity reason
1	Coca-Cola	Pepsi	High	Both use red/blue color schemes with circular/logotype elements; historic brand rivalry with similar retail contexts
2	Ferrari	Ford	High	Both feature horse logos; Ferrari’s prancing horse vs Ford Mustang’s galloping horse are frequently confused at a glance
3	Texaco	Shell	High	Both are petroleum brands using red/yellow/red color palettes with star/shell motifs; Texaco’s star and Shell’s scallop shape cause quick misidentification
4	FedEx	DHL	Medium-High	Both are shipping/logistics companies with bold sans-serif typography in purple/red/yellow color families
5	Stella Artois	Carlsberg	Medium-High	Both are European beer brands with ornate crest-style logos and similar gold/red/white color schemes
6	Adidas	Nvidia	Medium	Both use oblique striped/swoosh designs; Adidas three stripes and Nvidia’s green eye have been shown to cause spurious correlations in vision models
7	Heineken	Guiness	Medium	Both are dark beer brands with prominent red star elements and green/red/gold color palettes
8	BMW	Ford	Medium	Both automotive brands with blue/white roundels; BMW’s circular logo vs Ford’s blue oval create shape-based confusion
9	UPS	DHL	Medium-Low	Both are brown/gold/yellow delivery brands with shield/rectangle logo shapes; UPS’ brown shield vs DHL’s red/yellow rectangle
10	Paulaner	Erdinger	Medium-Low	Both are German wheat beer brands with similar Bavarian blue/white diamond patterns and ornate typography

### Ablation study

This ablation study evaluates the contribution of each component in LogoXpertNet across three datasets, FlickrLogos-32, BelgaLogos, and WebLogo-2M. Table 9 gives the results of the proposed model with ablation study. Starting with only the MobileNetV3 backbone, accuracy ranged from 88.70% to 89.51% across datasets. The addition of the CLFF module improved accuracy by 2–3 percentage points, while incorporating the HSE-SAB further increased performance by an additional 1–2 percentage points. The proposed model, which also includes the FA-CBAM, achieved near-perfect results: 99.89% accuracy on FlickrLogos-32, 99.83% on BelgaLogos, and 99.92% on WebLogo-2M, with similarly high precision, recall, F1 score, and AUC values and low standard deviations.

**Figure 12:**
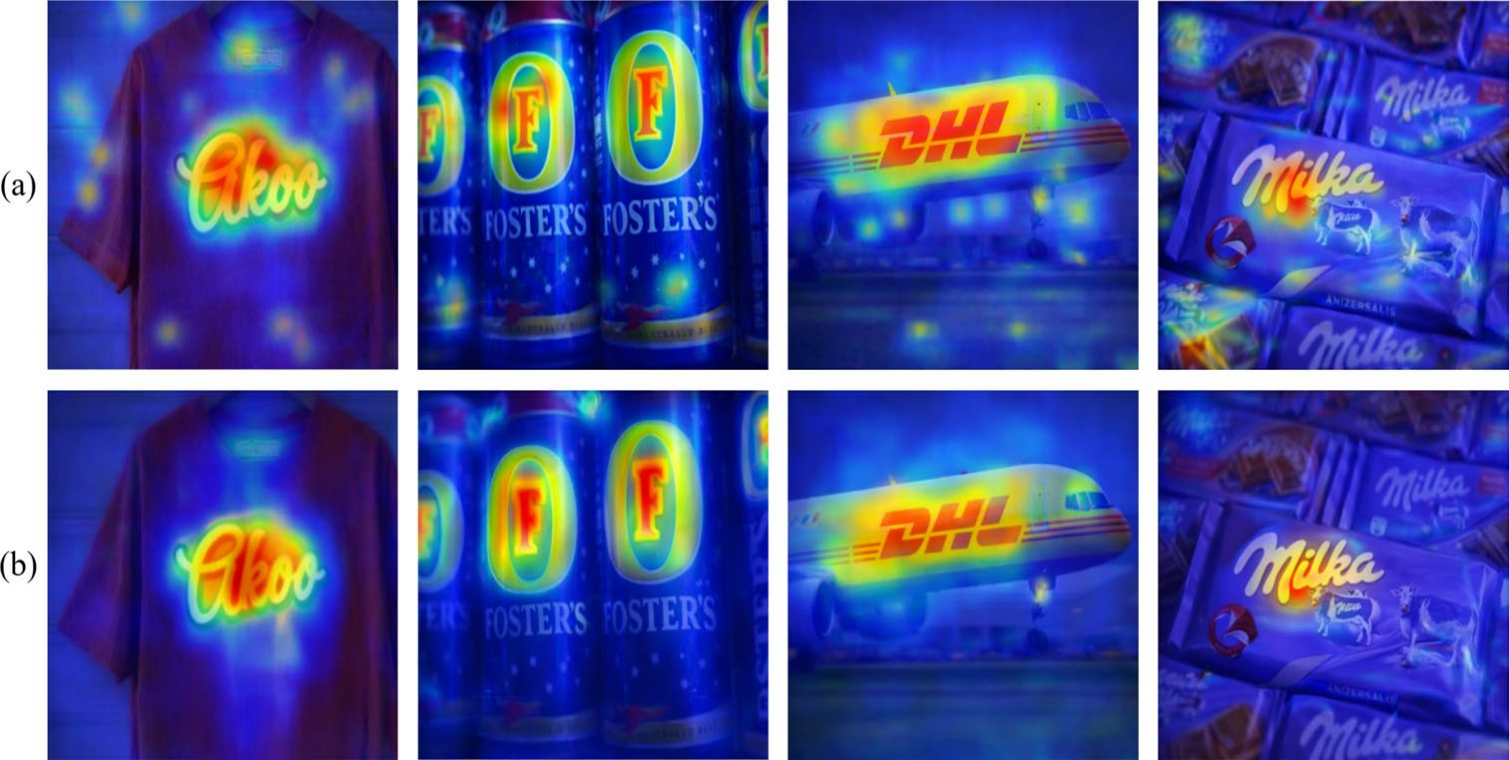
The visualization of feature maps for attention maps (**a**) HSE-SAB and (**b**) FA-CBAM.

The consistent improvements observed with each added component suggest that the proposed fusion and attention mechanisms contribute meaningfully beyond the MobileNetV3 backbone alone. The final LogoXpertNet configuration performs best among the evaluated ablated variants on the benchmark datasets considered here. Nevertheless, because the final reported values are very high, these gains should be interpreted as evidence of improved performance under the present benchmark protocol rather than definitive proof of universal robustness across all logo-recognition settings. The ablation results therefore support the usefulness of feature fusion and attention in this task while still leaving room for broader validation under more challenging cross-domain conditions. Figure 12 shows the attention feature maps of the proposed approach with different attention modules.

**Table 9:** Quantitative results of the LogoXpertNet with ablation study.

Dataset	MobileNetv3	CLFF	HSE-SAB	FA-CBAM	Accuracy (%)	Precision (%)	Recall (%)	F1 Score (%)	AUC (%)
FlickrLogos-32	✓	✗	✗	✗	89.51 [89.31, 89.71]	93.26 [93.10, 93.42]	94.75 [94.57, 94.93]	93.72 [93.48, 93.96]	95.65 [95.36, 95.94]
	✓	✓	✗	✗	92.80 [92.64, 92.96]	94.69 [94.55, 94.83]	93.87 [93.75, 93.99]	93.11 [92.91, 93.31]	96.60 [96.36, 96.84]
	✓	✓	✓	✗	94.27 [94.17, 94.37]	97.31 [97.19, 97.43]	96.91 [96.83, 96.99]	95.99 [95.83, 96.15]	97.23 [97.05, 97.41]
	✓	✓	✓	✓	**99.89 [99.85, 99.93]**	**99.96 [99.90, 100.02]**	**99.92 [99.90, 99.94]**	**99.93 [99.85, 100.01]**	**99.98 [99.94, 100.02]**
BelgaLogos	✓	✗	✗	✗	88.90 [88.66, 89.14]	92.50 [92.28, 92.72]	93.80 [93.62, 93.98]	92.70 [92.45, 92.95]	94.50 [94.23, 94.77]
	✓	✓	✗	✗	91.75 [91.57, 91.93]	93.85 [93.69, 94.01]	92.70 [92.56, 92.84]	92.60 [92.40, 92.80]	95.40 [95.18, 95.62]
	✓	✓	✓	✗	93.50 [93.36, 93.64]	96.50 [96.38, 96.62]	95.90 [95.80, 96.00]	94.85 [94.67, 95.03]	96.20 [96.04, 96.36]
	✓	✓	✓	✓	**99.83 [99.77, 99.89]**	**99.94 [99.90, 99.98]**	**99.93 [99.91, 99.95]**	**99.92 [99.88, 99.96]**	**99.94 [99.92, 99.96]**
WebLogo-2M	✓	✗	✗	✗	88.70 [88.48, 88.92]	91.80 [91.60, 92.00]	93.20 [93.02, 93.38]	91.95 [91.71, 92.19]	94.10 [93.85, 94.35]
	✓	✓	✗	✗	91.60 [91.42, 91.78]	93.25 [93.09, 93.41]	92.10 [91.96, 92.24]	92.05 [91.85, 92.25]	95.00 [94.78, 95.22]
	✓	✓	✓	✗	93.85 [93.73, 93.97]	96.20 [96.10, 96.30]	95.80 [95.72, 95.88]	94.70 [94.54, 94.86]	96.50 [96.36, 96.64]
	✓	✓	✓	✓	**99.92 [99.88, 99.96]**	**99.97 [99.85, 100.09]**	**99.94 [99.92, 99.96]**	**99.92 [99.88, 99.96]**	**99.95 [99.89, 100.01]**

Significant values are in bold.

## Conclusion

This study shows that LogoXpertNet is an effective lightweight framework for benchmark logo classification, combining cross-layer feature fusion, hierarchical attention, and frequency-aware refinement within an efficient MobileNetV3-based design. The model achieved strong results on FlickrLogos-32, BelgaLogos, and WebLogo-2M while maintaining practical computational efficiency. At the same time, because the reported accuracies are close to benchmark saturation, these findings should be interpreted with caution and in light of dataset difficulty, split construction, and metric definition. The present results therefore support the promise of LogoXpertNet on the evaluated benchmarks, while broader validation under more challenging real-world conditions remains necessary.

All efficiency evaluations are reported for an input resolution of 224 × 224 pixels under an image-level classification setting. While higher-resolution inputs may be required for extremely small or low-resolution logos, the computational complexity of LogoXpertNet scales linearly with input size due to its lightweight MobileNetV3 backbone. Similarly, the number of output classes affects only the final classification layer, resulting in negligible impact on inference speed. Handling extremely small logos through higher-resolution inputs or multi-scale inference is identified as an important direction for future work.

## Data Availability

This research employed the publicly accessible datasets, which can be downloaded from the following links: Flickr32-Logos: https://www.uni-augsburg.de/en/fakultaet/fai/informatik/prof/mmc/research/datensatze/flickrlogos/ BelgaLogos: https://www-sop.inria.fr/members/Alexis.Joly/BelgaLogos/BelgaLogos.html Web2M: https://github.com/pytorch/vision/tree/main/torchvision/datasets

## Code availability

The implementation code of the LogoXpertNet is available at https://github.com/Usman548/LogoXpertNet.
